# Cross-resistance patterns in SARS-CoV-2 against 3CL protease inhibitors

**DOI:** 10.1038/s41467-026-73444-y

**Published:** 2026-05-19

**Authors:** Janice Chithelen, David H. Lovett, Kevin Wang, Akari E. Torres Yanagisawa, Farah N. M. Caccin, Sho Iketani

**Affiliations:** 1 Aaron Diamond AIDS Research Center, Columbia University Vagelos College of Physicians and Surgeons, New York, NY USA; 2 Division of Infectious Diseases, Department of Medicine, Columbia University Vagelos College of Physicians and Surgeons, New York, NY USA

**Keywords:** SARS-CoV-2, Antiviral agents, Antimicrobial resistance

## Abstract

SARS-CoV-2 is now endemic, with infections commonplace. While much of the population now has immunity, some subsets remain at risk. For such individuals, small-molecule antivirals are the frontline treatment. However, studies have identified resistance-conferring mutations to these compounds, and there are now cases of resistant viruses emerging during treatment. These occurrences make clear the need to understand the resistance mechanisms for SARS-CoV-2 antivirals. Here, we report the pathways to resistance for atilotrelvir and simnotrelvir, two 3CL protease inhibitors used for COVID-19 treatment, and ibuzatrelvir, a compound in late-stage clinical development. Through high-throughput passaging, we reveal that resistance can readily arise, and that there is a large degree of overlap in the mutations which emerge. Moreover, viral inhibition assays demonstrate that there is not only strong cross-resistance between the emerged viruses against these three molecules, but also against two additional widely used antivirals, nirmatrelvir and ensitrelvir, as well. Cellular assays highlight S144A, E166A, and E166V as mediating broad resistance, with E166V having the strongest effects. These results have important clinical implications, including the need to carefully consider cross-resistance properties in salvage therapy and combination treatment, as well as emphasizing the need for the further development of SARS-CoV-2 antivirals with differing modalities.

## Abstract (web summary)

SARS-CoV-2 can develop resistance to antivirals. Here, the authors identify the pathways utilized by the virus to attain resistance to atilotrelvir, ibuzatrelvir, and simnotrelvir, revealing extensive cross-resistance among the selected viruses.

## Introduction

Though the COVID-19 pandemic is no longer considered a public health emergency and effective vaccines have been deployed, SARS-CoV-2 infections continue to be widespread, in part due to its continued evolution to evade immune responses^1^. While much of the population now have mild disease or remain asymptomatic upon infection, some subsets can develop much more severe disease. In particular, the millions of individuals that do not have a robust response to vaccination or infection, such as the immunocompromised population, are at risk, and certain subgroups are particularly at high risk^2–7^. For these and other vulnerable individuals, therapeutic interventions continue to be needed for management of infections and to protect them from severe disease.

Fortunately, efforts by the scientific community have resulted in the development of such treatment options. A number of monoclonal antibodies directed towards the viral spike glycoprotein, the protein mediating entry, were identified and found to demonstrate clinical efficacy, thereby resulting in their authorization for usage in patients. However, antibody resistance has emerged as SARS-CoV-2 has evolved, and the effectiveness of those originally approved have been compromised with the accumulation of mutations in the spike (reviewed in ref. ^8^).

Several direct-acting antiviral inhibitors remain available in their stead. The three available in the United States are remdesivir^9^ (Veklury), molnupiravir^10,11^ (Lagevrio), and nirmatrelvir^12,13^ (co-administered with ritonavir as Paxlovid). The first two target the RNA-dependent RNA polymerase (RdRp) and nirmatrelvir targets the 3CL protease (3CL^pro^; also known as nsp5 (nonstructural protein 5) or Main protease (M^pro^)), both essential viral proteins. In other countries, additional 3CL^pro^ inhibitors have been authorized or approved for clinical use: atilotrelvir^14,15^ (with ritonavir), ensitrelvir^16–18^, leritrelvir^19,20^, and simnotrelvir^21,22^ (with ritonavir) (Supplementary Fig. 1). These drugs have demonstrated clinical benefits and continue to be routinely employed as frontline therapies for COVID-19, although careful consideration of potential contraindications as well as the possibility of side effects are needed, which can limit their usage^23,24^. Accordingly, further second-generation 3CL^pro^ inhibitors are in development and are actively under clinical investigation^25–27^.

Yet, that is not to say that resistance cannot emerge for these small molecules. Indeed, given the history of viruses successfully evading antiviral therapies, it would be wise to expect that this would occur. Previous studies by our group and others utilizing viral passaging techniques have identified in vitro alterations which confer resistance to remdesivir^28–31^, nirmatrelvir^32–36^, and ensitrelvir^32,37^, while retaining sufficient fitness for viral growth. Many other experimental strategies have supported these observations and affirmed this approach^8^. Concerningly, for all three molecules, the mutations identified in these studies have now been reported in COVID-19 patients undergoing treatment, and have at times been associated with decreased treatment efficacy^36,38–50^. There is therefore a clear and pressing need to understand the resistance mechanisms for the clinically employed inhibitors to ensure appropriate clinical management and surveillance. Here, towards such an effort, we report the in vitro pathways for SARS-CoV-2 resistance to atilotrelvir and simnotrelvir, as well as a second-generation 3CL^pro^ inhibitor, ibuzatrelvir, which is currently in Phase 3 clinical trials^25,26^. Through large-scale passaging complemented with viral and cell-based assays, we reveal that there is significant overlap of resistance-associated residues, and consequent cross-resistance among current clinical 3CL^pro^ inhibitors.

## Results

### Overlapping pathways to resistance to 3CL^pro^ inhibitors

To safely assess SARS-CoV-2 resistance pathways to inhibitors, we first generated a recombinant SARS-CoV-2 strain with ORF3a and ORF7a double deletions, as it had been previously reported that such deletions would strongly attenuate the virus, particularly in vivo^51,52^. We successfully recovered an attenuated wild-type virus with these double deletions (SARS-CoV-2-ΔORF3a-ΔORF7a; USA-WA1/2020 backbone, GenBank accession no. MN985325.1)^53^, without any other additional mutations, through the use of circular polymerase extension reaction (CPER)^54–56^ (see Methods for details).

We first confirmed that this attenuated virus could be used for resistance studies by examining the inhibition of this virus in comparison to a wild-type virus without deletions (Supplementary Fig. 2). Similar inhibition profiles were observed by all tested 3CL^pro^ inhibitors, and only nominal differences were seen in IC_50_ values (1.34 to 1.48-fold), suggesting that the attenuated virus could recapitulate wild-type SARS-CoV-2 inhibition.

**Fig. 1:**
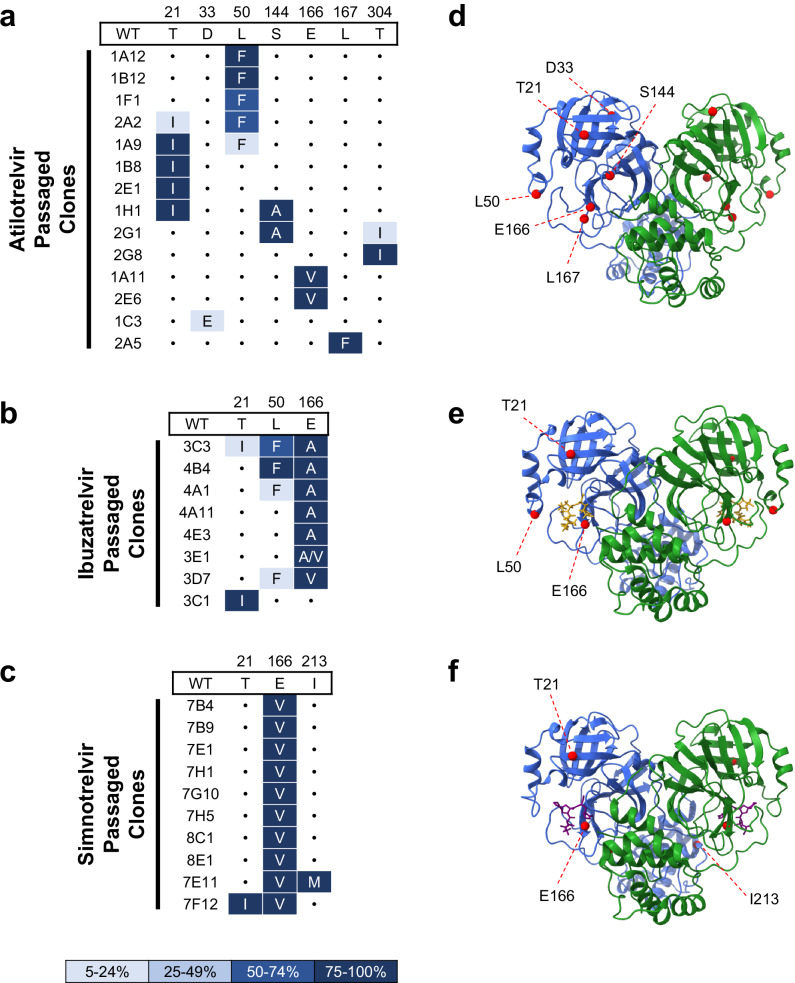
Emergent mutations during passaging for resistance to 3CL^pro^ inhibitors. Mutations observed in nsp5 after passaging for 12 or 14 passages against atilotrelvir (**a**), ibuzatrelvir (**b**), and simnotrelvir (**c**). Only residues mutated in the terminal passage for each inhibitor are shown. Each well represents an independently passaged replicate. Dots indicate WT at that residue in that clone. Mutations are colored according to frequency (see Supplementary Data 2 for frequencies of each mutation in each clone). R222P is not shown as it was observed in untreated passaged viruses, suggesting it is a nonspecific cell-culture adaptation (Supplementary Data 3). A/V denotes a mixture of A and V. Residues mutated after drug passaging, overlaid onto 3CL^pro^ structures, for atilotrelvir (**d**), ibuzatrelvir (**e**), and simnotrelvir (**f**). The Cα of each mutated residue is denoted by a red sphere. Protomer A is shown in royal blue and protomer B is shown in forest green. For atilotrelvir (**d**), as a compound-bound structure was not available, a ligand-free 3CL^pro^ was used instead for illustrative purposes. Note however that it has been previously modeled to likely bind in the same manner as nirmatrelvir^15^. Ibuzatrelvir is colored goldenrod in **e** and simnotrelvir is colored purple in (**f**). 3CL^pro^ complexes were downloaded from PDB under accession codes 7JST^83^ (ligand-free, **d**), 8V4U^25^ (ibuzatrelvir, **e**), and 8IGX^22^ (simnotrelvir, **f**). Note that T304 is not shown in **d** as this residue was not modeled in this structure. Abbreviations: WT wild-type.

We then used this virus to conduct high-throughput passaging against atilotrelvir, ibuzatrelvir, and simnotrelvir (Fig. 1, Supplementary Fig. 3, 4, 5, and 6, and Supplementary Data 1, 2, and 3). Passaging was carried out with 180 replicates for each compound for 12 or 14 passages, employing an improved version of a scalable strategy that we had previously developed to identify nirmatrelvir resistance^33^. This usage of significantly more replicates than conventional passaging strategies can provide a more complete picture of the pathways to resistance as well as expedite the selection process (see Methods for details). Infected replicates at the conclusion of passaging, as well as their respective stored samples from earlier passages, were then assessed by next-generation sequencing to identify resistance-associated substitutions.

Distinct patterns of mutations were observed to arise against each of the compounds. Alterations were observed both proximal and distal to the substrate-binding site targeted by all three drugs. Atilotrelvir had the most mutational diversity among sequenced clones, suggesting that a multitude of pathways exist to achieve resistance against this compound (Fig. 1a, d and Supplementary Fig. 4). Several common groups of mutations were seen however, with T21I and L50F (both 35.7%; 5/14 clones) as the highest occurring mutations, and S144A, E166V, and T304I each also observed in 14.3% of the clones (2/14). All of these mutations frequently occurred in pairs, potentially indicative of synergistic and/or compensatory mechanisms.

In contrast, select mutations dominated the sequenced clones for ibuzatrelvir and simnotrelvir, pointing towards specific routes to resistance for these compounds. Mutations at E166 were seen in 87.5% of clones (7/8) for ibuzatrelvir, with all but one being E166A (85.7%; 6/7) (Fig. 1b, e and Supplementary Fig. 5). Over half of these E166 mutants were complemented by L50F. Simnotrelvir passaging cleanly had E166V in all clones (100%; 10/10), clearly emphasizing the significance of this substitution for this drug (Fig. 1c, f and Supplementary Fig. 6). These results demonstrate that resistance to all three 3CL^pro^ inhibitors is readily achievable by SARS-CoV-2 in vitro, and highlight that there are specific mutations which consistently arise for each drug.

**Fig. 2:**
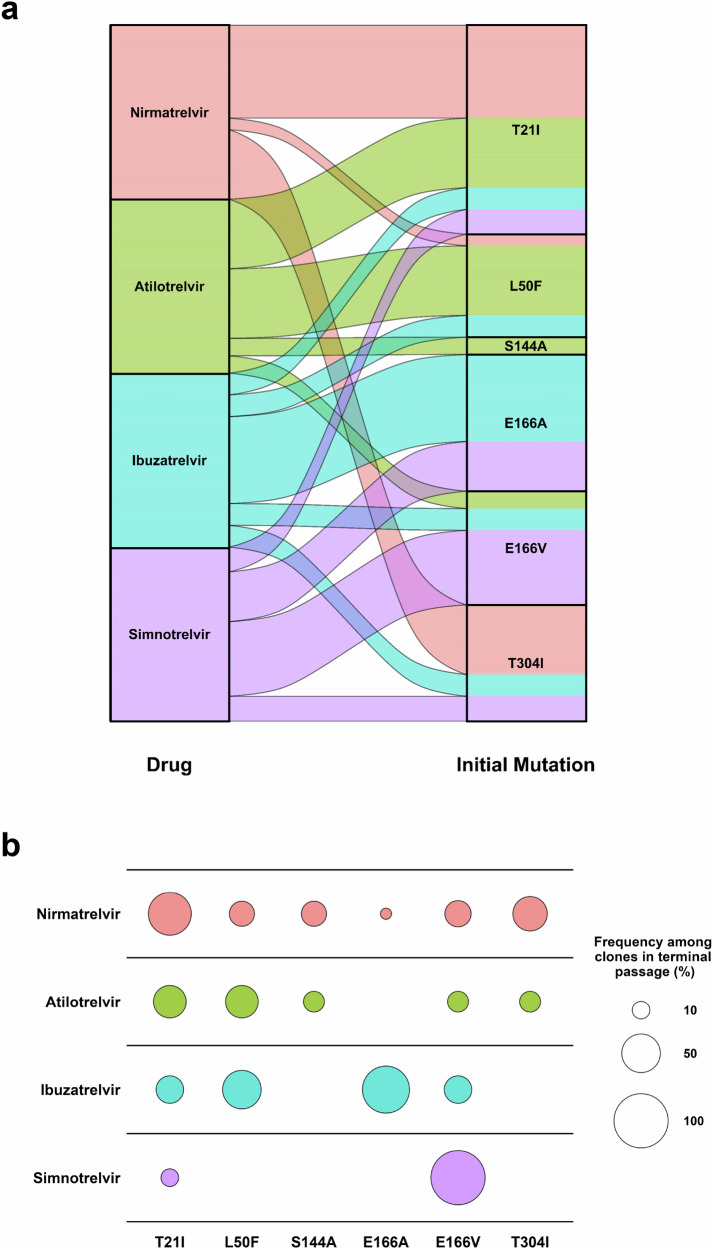
Pathway analyses of resistance to 3CL^pro^ inhibitors. **a** Mutations which first arose during passaging against nirmatrelvir, atilotrelvir, ibuzatrelvir, and simnotrelvir, shown as an alluvial plot^78^. Mutational trajectories for each drug were normalized to account for the different numbers of observed transitions. See Methods for details and Supplementary Data 4 for datapoints used. **b** Mutations found across clones at the terminal passage. See Supplementary Data 5 for datapoints used. For both analyses, only T21I, L50F, S144A, E166A, E166V, and T304I were included, as they were the most frequently emerging mutations. Data for nirmatrelvir is from our previous report^33^.

In our analysis, we noticed that six mutations—T21I, L50F, S144A, E166A, E166V, and T304I—were repeatedly observed across our passaging experiments. Given the potential clinical implications of such overlapping resistance profiles, we delved deeper into the pathways to resistance for these 3CL^pro^ inhibitors, as well as for nirmatrelvir from our previous study^33^. We first considered which mutations initially arose among the clones against each of the compounds (Fig. 2a and Supplementary Data 4). We assessed transition events among all clones from passaging, identifying those that we could confidently place for the aforementioned six mutations (see Methods for details). All mutations occurred at similar frequencies across the four compounds (12.5% to 20% of all transitions), with the exception of T21I and S144A, which were more common (35%) or rare (2.5%), respectively. Apart from S144A, the remaining five mutations were observed to overlap as initial transitions for at least two drugs, with T21I emerging against all four 3CL^pro^ inhibitors. L50F, E166V, and T304I were initial events against three out of four inhibitors.

We next examined the mutations harbored by clones in the terminal passages, against focusing on the above six mutations of high occurrence (Fig. 2b and Supplementary Data 5). A strong overlap in terminal resistance profiles was seen across the four compounds, with each of the inhibitors sharing two or more mutations with all other inhibitors. Each of the six mutations that were included in our analysis were observed to occur for multiple drugs. T21I and E166V were found across all four inhibitors, albeit at varying frequencies. Collectively, these data illustrate that there is a high degree of convergence in routes to resistance to these four clinical 3CL^pro^ inhibitors by SARS-CoV-2.

### Cross-resistance among 3CL^pro^ inhibitors

One major corollary of the observed overlapping pathways to resistance is that the viruses which emerge under pressure of one drug may potentially be cross-resistant to another. To test for this possibility, we selected a set of five diverse clones from the terminal passages of each of the passaging experiments for further study. These viruses represented a number of different combinations of mutations of interest, and included those specific mutations highlighted in the prior pathway analyses (Fig. 2).

The five passaged viruses were expanded to have sufficient material, and then sequenced to confirm that they had retained their nsp5 mutations. All had analogous mutations to before expansion, with the exception of P12-2G1, which had a low frequency of T304I that was lost after expansion (Supplementary Data 6).

As these viruses had thus far been passaged only in 293T-ACE2-TMPRSS2-mCherry cells, we examined whether these viruses could grow in a human lung cell line. Growth assays in A549-ACE2 cells demonstrated that the passaged viruses could readily grow in this cell line as well, achieving greater titer than the original wild-type virus (with ORF3a and ORF7a double deletions), which had not been passaged (Supplementary Fig. 7). Such a difference may reflect adaptation to in vitro conditions, which we also observed in our prior study^33^.

**Fig. 3:**
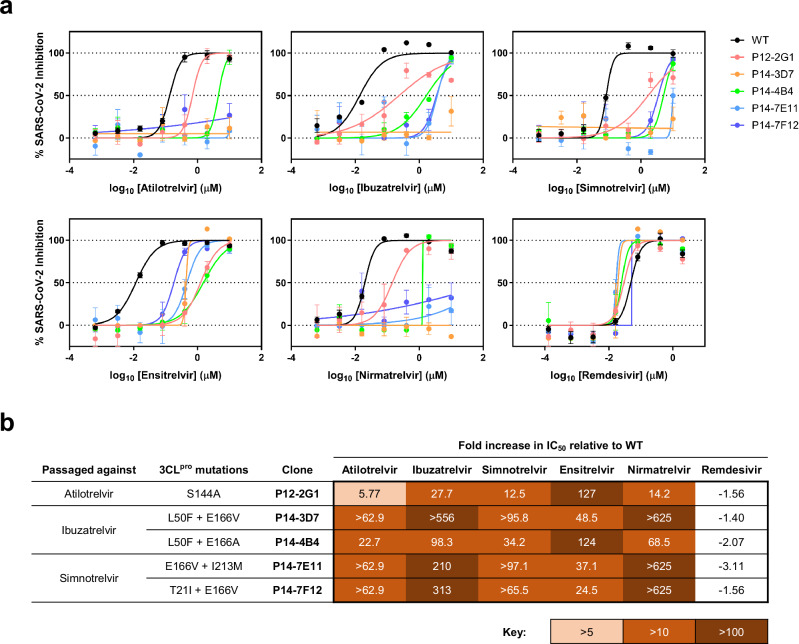
Cross-resistance of passaged viruses. **a** Inhibition curves of wild-type or passaged viruses by 3CL^pro^ inhibitors or remdesivir. Representative curves from a single experiment from two biologically independent experiments are shown. Error bars denote mean ± SEM of three technical replicates. **b** Fold increase in mean IC_50_ values relative to wild-type inhibition. Passaged viruses were first sequenced to confirm their 3CL^pro^ mutations, which are listed in the second column (see Supplementary Data 6 for mutation frequencies). All clones are from the terminal passages against their respective compounds. Source data are provided as a Source Data file.

Each of the viruses was then tested for inhibition by atilotrelvir, ibuzatrelvir, and simnotrelvir to examine if they displayed any cross-resistance (Fig. 3 and Supplementary Fig. 8). Ensitrelvir and nirmatrelvir were also included as clinically relevant 3CL protease inhibitors. Remdesivir was tested as a control, as it does not target the 3CL^pro^ and targets the RdRp.

The viruses were first examined for their inhibition by the respective compounds that they were passaged against, to affirm that the passaging resulted in specifically resistant viruses. Resistance to the passaging compounds ranged from mild (5.77-fold; P12-2G1 to atilotrelvir) to very strong (>556-fold; P14-3D7 to ibuzatrelvir), supporting the employed strategy. No resistance was seen to remdesivir by any of the viruses, suggesting that resistance to the 3CL^pro^ inhibitors was specific and not a general viral phenotype.

We next investigated the inhibition of each virus in all pairwise combinations to the other 3CL^pro^ inhibitors. Extensive, broad cross-resistance was evident across the tested viruses, with moderate (10 to 100-fold) and strong (>100-fold) resistance by all of the viruses against all of the compounds, with the sole exception of P12-2G1 to atilotrelvir, which had mild resistance (5.77-fold). For four of the drugs—atilotrelvir, ibuzatrelvir, simnotrelvir, and nirmatrelvir—viruses with E166V were consistently the most resistant (P14-3D7, P14-7E11, and P14-7F12; >62.9 to >625-fold), followed by the viruses with L50F + E166A (P14-4B4; 22.7 to 98.3-fold), then S144A (P12-2G1; 5.77 to 27.7-fold). In contrast, E166V conferred moderate resistance to ensitrelvir (24.5 to 48.5-fold), and the strongest resistance towards this molecule was observed with S144A (P12-2G1; 127-fold) and L50F + E166A (P14-4B4; 124-fold).

These resistance profiles indicate that these 3CL^pro^ inhibitors can be classified into two groups on the basis of how they are affected by E166V, E166A, and S144A. The first and predominant group were those compounds which were affected most by E166V above the other mutations; this group consists of atilotrelvir, ibuzatrelvir, simnotrelvir, and nirmatrelvir. The second group consists of ensitrelvir alone, which was most susceptible to the resistance of S144A. In addition, this group appears to be affected more by E166A than E166V. This classification scheme is in line with the compound chemical structures, binding modes, and residue interactions (Supplementary Fig. 1 and 9). Altogether, these data underscore the strong likelihood that viruses emerging under the pressure of one 3CL^pro^ inhibitor will demonstrate cross-resistance to others.

### Resistance-conferring 3CL^pro^ mutations

**Fig. 4:**
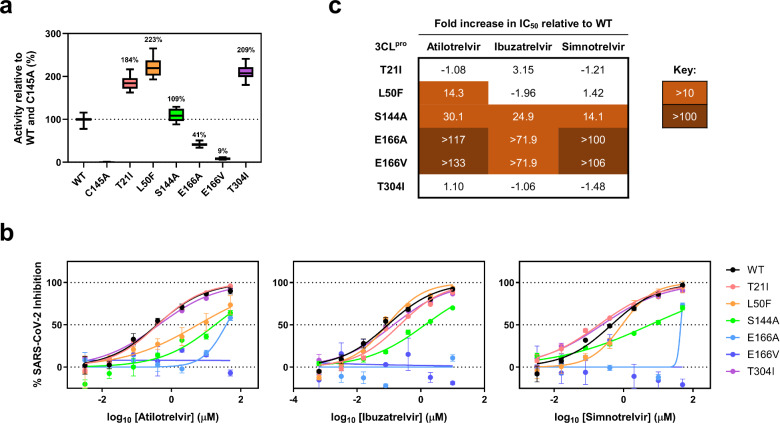
Activity and resistance of individual 3CL^pro^ mutants. **a** Activity of wild-type or mutant 3CL proteases in the FlipGFP cellular reporter assay using nsp4-nsp5-nsp6 fusion proteins (see Methods for details). Representative results from a single experiment from two biologically independent experiments are shown. Data are shown as a box and whiskers plot with whiskers from the minimum to maximum values for 18 technical replicates. The middle line of the box is the median and the box bounds are the 25th and 75th percentiles. Values above boxes denote means. **b** Inhibition curves of wild-type or mutant 3CL proteases by atilotrelvir, ibuzatrelvir, and simnotrelvir. Representative curves from a single experiment from two biologically independent experiments are shown. Error bars denote mean ± SEM of three technical replicates. **c** Fold increase in mean IC_50_ values relative to wild-type inhibition. Source data are provided as a Source Data file.

To validate in an orthogonal assay that specific 3CL^pro^ mutations conferred the observed resistance to atilotrelvir, ibuzatrelvir, and simnotrelvir, we utilized a cellular assay with a protease-activated fluorogenic reporter (FlipGFP)^57–60^. Optimization of the assay design allowed for mutant 3CL^pro^s to be evaluated (Fig. 4a, Supplementary Fig. 10, and Methods). We tested the six major resistance-associated mutations in this assay (T21I, L50F, S144A, E166A, E166V, and T304I). In line with prior reports^61–63^, T21I and L50F increased protease activity relative to wild-type (223% and 184%, respectively), whereas E166A and E166V hampered the 3CL^pro^ (41% and 9%, respectively), with E166V having a more negative effect than E166A (Fig. 4a).

Each of the 3CL^pro^s was then tested for inhibition by atilotrelvir, ibuzatrelvir, and simnotrelvir (Fig. 4b, c, and Supplementary Fig. 11). T21I and T304I did not confer notable resistance, and L50F conferred moderate resistance only to atilotrelvir (14.3-fold). Broad resistance to all three inhibitors was observed with S144A, E166A, and E166V. Congruent to the viral assays, the strongest resistance was seen with E166V (>71.9 to >133-fold) followed by E166A (>71.9 to >117-fold). S144A resulted in moderate resistance (14.1 to 30.1-fold). These results affirm that these mutations confer broad cross-resistance to these 3CL^pro^ inhibitors.

Finally, we examined whether these mutations have been increasing in frequency over the years, as the inhibitors have become available and regularly used (Supplementary Fig. 12). All of the six 3CL^pro^ mutations were seen in clinical sequences deposited to GISAID^64^, indicating that they are viable as viruses in patients. However, their overall frequencies remained low and we did not note any appreciable trends for these mutations, suggesting that the circulation of viruses with these substitutions is limited.

## Discussion

The effectiveness of the therapeutics that have been developed for COVID-19 has been a testament to the efforts of the scientific community to combat this disease. However, the potential for the emergence of antiviral resistance remains an ever-present concern, making further discovery of additional inhibitors continue to be needed. Indeed, current SARS-CoV-2 variants exhibit resistance to every clinically-utilized antibody, making small molecules the necessary treatment of choice^8^. Yet, viruses with resistance-associated substitutions have been identified in patients receiving remdesivir, nirmatrelvir, and ensitrelvir, suggesting that they too, require careful consideration and administration, particularly in high-risk cases involving extended courses of treatment^8,36,38–50^. These experiences have underscored the importance of understanding the resistance profiles of SARS-CoV-2 antivirals. Herein, we have delineated the resistance pathways for atilotrelvir, ibuzatrelvir, and simnotrelvir (Figs. 1 and 2), as well as comprehensively evaluated the resistance profile of the viruses and mutations selected under the pressure of these drugs (Figs. 3 and 4). These results demonstrate that resistance can be readily achieved by SARS-CoV-2-against these compounds, and that there is extensive cross-resistance among the viruses which emerge.

Using our high-throughput passaging strategy, we observed that specific mutation profiles arose against each of the molecules (Fig. 1), albeit with significantly overlapping pathways to achieve this resistance (Fig. 2). Atilotrelvir predominantly had T21I and L50F arise, whereas ibuzatrelvir and simnotrelvir had E166A and E166V, respectively. A set of six mutations—T21I, L50F, S144A, E166A, E166V, and T304I—was commonly observed to emerge against these three inhibitors as well as nirmatrelvir. As would be predicted by the convergence in mutations arising during passaging, inhibition assays with passaged viruses demonstrated strong cross-resistance across the three compounds, as well as against nirmatrelvir and ensitrelvir (Fig. 3). Cellular assays demonstrated that this cross-resistance was mediated by S144A, E166A, and E166V (Fig. 4). The inhibitors could be classified into two groups based on the resistance they faced against several mutations, with E166V defining the largest group, consisting of atilotrelvir, ibuzatrelvir, simnotrelvir, and nirmatrelvir. E166V conferred the strongest resistance to each of these drugs. Ensitrelvir was unique, as it was affected more by E166A than E166V, and most susceptible to S144A out of the mutations. This is likely due to the similarity in chemical structures and binding modes of the compounds (Supplementary Figs. 1 and 9).

While we have extensively studied the resistance pathways and inhibition profiles of these three antivirals, it is clear that additional structural and biochemical analyses will be required to intimately dissect the mechanisms of resistance by each of the mutations against each of these compounds. However, some hints are provided by studies conducted thus far on nirmatrelvir resistance. T21I and L50F, which were commonly observed in the passaging experiments (Figs. 1 and 2), have been shown to provide a fitness benefit for the virus, counteracting the fitness defects associated with other resistance-associated mutations^33^. This appears to be in part due to the heightened enzymatic activity conferred by these substitutions (Fig. 4a)^61–63^. Both E166 and S144 are critical residues within the S1 subsite of the substrate binding region, and structural studies have indicated that the E166A/V and S144A mutations heavily disrupt this pocket, affecting binding to nirmatrelvir. Yet, these alterations also interfere with binding to the substrate, and in the case of E166A/V, also affect the dimerization capacity of the 3CL^pro^, leading to the fitness losses^33,61–63^. It will have to be ascertained whether such mechanisms are common to atilotrelvir, ibuzatrelvir, and simnotrelvir as well.

Viral passaging has proven to be prescient of the resistance-associated substitutions which arise in vivo for SARS-CoV-2, and we therefore believe that there are several important clinical considerations arising from this present work. First, the mutations identified as conferring resistance to each of the compounds should be surveilled as these antivirals are administered, particularly during the extended and repeated courses that are commonly given to immunocompromised individuals that can develop persistent infections^38,40–45,49,50^. Second, in the case that such resistance does arise and there is treatment failure, if salvage therapy is to be conducted using another 3CL^pro^ inhibitor, it should not be with a compound that overlaps in resistance profile and has cross-resistance to the emerged virus. Finally, as we consider the potential use of combination therapies for high-risk populations to increase their efficacy and heighten the barriers to resistance, the 3CL^pro^ inhibitors, which overlap in their pathways to resistance, would be expected to largely have additive effects, rather than synergism, and effective combinations would need to be identified. Given that COVID-19 is now an endemic disease and infections routinely occur, high-risk individuals will need options to protect them. We suggest that clinical trials focused on identifying salvage therapy options as well as synergistic multi-drug regimens in these populations are needed to inform appropriate clinical guidance. Welcomingly, such ideas are now being considered and developed^65–67^.

As we purposely utilized an attenuated backbone that is compromised in vivo for biosafety, we could not validate our findings in vivo. However, we note that for nirmatrelvir and ensitrelvir, it has been observed that the relative fitness and resistance of resistant viruses have largely been congruent with in vitro data when tested in vivo^37,68,69^. It should also be pointed out that we have only conducted the passaging in one cell line, and that we have used the original USA-WA1/2020 strain as the base of our experiments. It is possible that some of the results may differ in an alternative cell type, or with the current variants, which harbor P132H in the 3CL^pro^ gene as well as numerous additional mutations elsewhere in their genomes. Other studies have noted that the fitness impact and emergence of 3CL^pro^ mutations can be impacted by the SARS-CoV-2 backbone^34,70^. Additional studies will be needed to investigate these prospects.

Fortunately, resistance to 3CL^pro^ inhibitors has not currently become widespread (Supplementary Fig. 12). Yet, our results reveal that there are critical weaknesses in our current arsenal of COVID-19 drugs. Importantly, in the event that a new coronavirus emerges in the future, it is likely that these antivirals will be our first line of defense. For protecting high-risk individuals in the present and for future pandemic preparedness, the continued development of next-generation inhibitors which differ from current modalities is needed.

## Methods

### Biosafety and biocontainment

To mitigate potential biosafety risks, all recombinant SARS-CoV-2 production, passaging, and infections were conducted by trained and vaccinated personnel in BSL-3 laboratories at Columbia University Irving Medical Center under procedures and guidelines approved by the Columbia University Institutional Biosafety Committee (IBC). Experiments involving recombinant viruses were only conducted utilizing an attenuated SARS-CoV-2 strain with double ORF3a and ORF7a deletions^51,52^, as approved by NIAID prior to the initiation of the research. This strain is heavily attenuated and deficient in replication, particularly in vivo. The benefits of conducting the work are to inform appropriate usage of the compounds which were studied, as well as to provide guidance for future drug development.

### Compounds

Atilotrelvir, ibuzatrelvir, and simnotrelvir were purchased from MedChemExpress. Nirmatrelvir was purchased from Aobious. Ensitrelvir was purchased from Glixx Laboratories. Remdesivir was purchased from Selleckchem. Compounds were resuspended to 100 mM in DMSO and stored at −20 °C until use.

### Cells

293T-ACE2-TMPRSS2-mCherry cells (HEK293T cells overexpressing human ACE2 and TMPRSS2) were obtained from BEI Resources (Catalog # NR-55293, gift of C. Weiss). Vero E6-TMPRSS2-T2A-ACE2 cells (Vero E6 cells overexpressing human TMPRSS2 and ACE2) were obtained from BEI Resources (Catalog #NR-54970, gift of B. Graham). Lenti-X™ 293T cells were purchased from Takara Bio (Catalog #632180). A549 cells were purchased from ATCC (Catalog #CCL-185). A549-ACE2 cells (A549 cells overexpressing human ACE2) were generated by transduction of A549 cells with lentivirus packaged using pLEX307-ACE2-blast (Addgene plasmid #158449), pMD2.G (Addgene plasmid #12259, gift of D. Trono), and psPAX2 (Addgene plasmid #12260, gift of D.r Trono), followed by selection with 5 µg/mL blasticidin. Cell morphology was visually confirmed prior to use and all cell lines tested mycoplasma negative. All cells were grown in complete medium (DMEM + 10% fetal bovine serum + penicillin/streptomycin) and maintained at 37 °C under 5% CO_2_.

### Production of attenuated SARS-CoV-2

Attenuated SARS-CoV-2 with double ORF3a and ORF7a deletions (SARS-CoV-2-ΔORF3a-ΔORF7a)^51,52^ was produced by circular polymerase extension reaction (CPER) as previously described, with modifications to improve viral recovery^54–56^. The SARS-CoV-2 genome sequence (USA-WA1/2020 strain, GenBank accession no. MN985325.1)^53^ was divided into 10 overlapping fragments as described^56^ (see Supplementary Data 7 for fragment sequences). ORF3a and ORF7a were deleted within fragments #9 and #10, respectively. The ORF3b gene found within ORF3a was not retained and was also deleted. In place of ORF7a, EGFP and NanoLuc separated by a T2A peptide were inserted, allowing for bicistronic expression of EGFP and NanoLuc^71^. Each of the fragments was then synthesized as two or three shorter fragments (Twist Biosciences), which were assembled together into the pUC57 vector (Thermo Fisher Scientific) by NEBuilder HiFi DNA Assembly (New England Biolabs) and amplified in NEB® Turbo *E. coli* (New England Biolabs). An eleventh linker fragment was also synthesised and cloned into pUC57, consisting of a hepatitis delta virus ribozyme (HDVr), SV40 poly(A) signal, RNA pol II transcriptional pause signal^55,72^, CMV enhancer, and CMV promoter. All sequences were verified by nanopore sequencing (Plasmidsaurus) prior to use.

Each fragment was then amplified from the individual plasmids by PCR by combining 0.8 µL of Platinum™ SuperFi™ II DNA Polymerase (Thermo Fisher Scientific), 8 µL of 5X SuperFi™ II Buffer, 0.8 µL of 10 mM dNTPs, 2 µL of 10 µM forward primer, 2 µL of 10 µM reverse primer, 10 ng of template, and H_2_O to 40 µL. Analogous primers to those previously described were used for amplification of each fragment^56^, with modifications to primers to amplify fragments #9 and #10 due to the introduced alterations (see Supplementary Data 7 for primer sequences). PCR was conducted in a thermocycler with the following conditions:

1. 98 °C, 30 s

2. 98 °C, 10 s

3. 60 °C, 10 s

4. 72 °C, 2 min

5. Return to step 2 for 34 additional cycles

6. 72 °C, 5 min

7. Hold at 10 °C

All fragments were then gel-purified (Zymo Research). CPER reactions were assembled by combining 0.1 pmol of each fragment, 2 µL of Platinum™ SuperFi™ II DNA Polymerase, 10 µL of 5X SuperFi™ II Buffer, 1 µL of 10 mM dNTPs, and H_2_O to 50 µL. CPER was conducted in a thermocycler with the following conditions:

1. 98 °C, 30 s

2. 98 °C, 10 s

3. 60 °C, 10 s

4. 72 °C, 16 min

5. Return to step 2 for 34 additional cycles

6. 72 °C, 16 min

7. Hold at 10 °C

While the CPER reaction was ongoing, 1.3 × 10^6^ 293T-ACE2-TMPRSS2-mCherry cells were seeded in a well of a 6-well plate. The following day, the CPER reaction was transfected into the cells by mixing all 50 µL of the CPER reaction with 250 µL Opti-MEM and 7.5 µL TransIT™-LT1 Reagent (Mirus Bio), incubation at RT for 15 min, and then adding the mixture onto the cells. Transfected cells were incubated at 37 °C for 72 h. On the second day, 3 × 10^6^ Vero E6-TMPRSS2-T2A-ACE2 cells was seeded in a T25 flask, allowing for overlay of the transfected 293T-ACE2-TMPRSS2-mCherry cells onto the Vero E6-TMPRSS2-T2A-ACE2 cells at 72 h post-transfection. Cells were incubated at 37 °C for an additional 6 days until cytopathic effects (CPE) were observed. The supernatant containing the virus was then collected, clarified by centrifugation, and then used to infect fresh cells in a larger volume to generate stocks. Stocks were frozen and stored at −80 °C until further use. Prior to use in experiments, a frozen aliquot was thawed and used for titration by the Reed-Muench method^73^, as well as sequence-verified by nanopore sequencing to contain no mutations other than the ORF3a and ORF7a deletions.

### In vitro selection for SARS-CoV-2 resistance

To identify resistance mechanisms, we adapted our previously reported strategy of high-throughput selection of SARS-CoV-2 resistance by blind volumetric passaging across a high number of replicates^33^ (Supplementary Fig. 3). In this paradigm, we conduct infections in a 96 well-plate, with each well serving as an independent replicate. These replicates are treated with drug and passaged to new cells every three days, with drug doses doubled every other passage. As each well contains only a limited number of cells, there is an implicit bottleneck effect, which allows for both major and minor mutations to be observed, rather than dominant mutations overtaking the pool. In addition, the selection of resistance is expedited due to the use of a large number of replicates, which helps to curtail the effects of the stochasticity that can be present in infections. It is also appealing that this passaging approach does not involve the potentially laborious need for quantitation of virus in each replicate prior to passaging, as is typically done in standard passaging approaches to determine which replicates at which doses are moved forward. We previously observed that this high-throughput approach could select for resistant viral populations in nearly half of the number of passages as more conventional passaging strategies^33^.

Passaging was conducted in 293T-ACE2-TMPRSS2-mCherry cells with 180 wells for each compound. Infections were initiated with 0.05 MOI of SARS-CoV-2-ΔORF3a-ΔORF7a and compounds were initially added at 90 nM (atilotrelvir), 30 nM (ibuzatrelvir), or 80 nM (simnotrelvir). The total volume of each well was 200 µL. Cells were seeded 24 h prior to infection at a density of 3 × 10^4^ cells/well. As controls, each plate contained three wells which were infected but untreated and three wells which were uninfected and untreated, and supernatant from these wells was also continuously passaged with the experimental wells.

Infections were allowed to proceed for 72 h, before 50 µL of the supernatant was transferred to fresh cells and treated with fresh drug. The drug dose was doubled every other passage. Passaging was conducted for 12 (atilotrelvir) or 14 total passages (ibuzatrelvir and simnotrelvir). The terminal dose was 2880 nM (atilotrelvir), 1920 nM (ibuzatrelvir), or 5120 nM (simnotrelvir). Supernatant was collected every fourth passage, as well as the terminal 14th passage for ibuzatrelvir and simnotrelvir, and stored at −80 °C for later sequencing. In addition, for these passages, CPE was quantified for all wells by CellTiter-Glo® 2.0 Cell Viability Assay (Promega) according to the manufacturer’s recommendations on a CLARIOstar Plus (BMG Labtech) instrument with SMART Control software (BMG Labtech, version 6.10).

### Sequencing of passaged SARS-CoV-2

A subset of the wells, which were found to be infected at the terminal passage, as determined by CPE, as well as all stored earlier passages of the analogous wells, were sequenced by next-generation sequencing to determine resistance-associated mutations. The terminal passage of four control wells, which were infected but untreated and were simultaneously passaged, was also sequenced. Viral RNA was extracted from 25 µL of the frozen supernatant using PureLink™ Viral RNA/DNA Mini Kit (Invitrogen) or E.Z.N.A.® Viral RNA Kit (Omega Bio-tek) according to the manufacturer’s recommendations. Amplicons spanning the nsp5 gene were prepared as previously described^74^ (dx.doi.org/10.17504/protocols.io.bwyppfvn), using LunaScript^®^ RT SuperMix Kit (New England Biolabs) for first-strand cDNA synthesis followed by PCR with Q5® High-Fidelity DNA Polymerase (New England Biolabs). Two separate PCR reactions were conducted for each sample using previously described primers^74,75^: (1) 5’-AGACACCTAAGTATAAGTTTGTTCGCA-3’ + 5’-GCCCACATGGAAATGGCTTGAT-3’, (2) 5’-TTTACCAGGAGTTTTCTGTGGTGT-3’ + 5’-TGGGCCTCATAGCACATTGGTA-3’. PCR products were then sequenced by nanopore sequencing (Plasmidsaurus) and analyzed by the wf-artic pipeline (version 1.1.0) in EPI2ME (Oxford Nanopore Technologies, version 5.1.2). Mutations were called with LoFreq^76^ (version 2.1.5) at a frequency threshold of 5% using the Wuhan-Hu-1 strain as reference (GenBank accession no. MN908947.3)^77^. Raw sequencing reads have been deposited to NCBI SRA under BioProject accession number PRJNA1308603 and accession numbers for each sample are shown in Supplementary Data 1. Frequencies of mutations in each sample are shown in Supplementary Data 2 and 3.

### Mutational analyses for passaged viruses

For the analysis of the initially occurring mutations against different drugs shown in Fig. 2a, the acquisition pathway of mutations for each replicate was determined as previously described, with modifications^33^. Only transitions resulting in T21I, L50F, S144A, E166A, E166V, or T304I were considered in this analysis, as these were the commonly observed mutations across the compounds. In addition, only the first transitions emerging from wild-type were included, with the exception of synonymous mutations and R222P, which was allowed as it was observed in untreated passaged virus, suggesting it is a nonspecific cell-culture adaptation. In cases of two or more mutations arising from wild-type between passages, if the pairwise frequencies summed to less than 100%, these transitions were excluded as it was not always clear if the mutations co-occurred in the same virus. In cases where the frequencies summed to greater than 100%, these mutations were assumed to co-occur in the same virus, and the mutation with higher frequency was treated as having arisen first. All observed transitions were included in the analysis. Nirmatrelvir passaging transitions were taken from our prior study^33^. After normalizing to account for the different number of transitions observed across drugs, the relative incidence of the first emerging mutations was plotted using ggalluvial^78^ (version 0.12.5) and ggplot2 (version 3.5.1) in R (version 4.4.1). The datapoints used in this analysis are shown in Supplementary Data 4.

For the frequency of mutations among replicates in the terminal passage shown in Fig. 2b, all datapoints for the terminal passages of all replicates as shown in Supplementary Data 2 were tabulated for the presence of T21I, L50F, S144A, E166A, E166V, or T304I. Nirmatrelvir passaging mutations were taken from our prior study^33^. No cutoff was applied and mixed populations of E166A and E166V were counted for both mutations. The plot was generated using ggplot2 (version 3.5.1) in R (version 4.4.1). The tabulated datapoints used in this analysis are shown in Supplementary Data 5.

### Expansion of passaged viruses

To conduct further assays with passaged SARS-CoV-2, clones of interest were first expanded to have a sufficient amount of virus. Approximately 3 × 10^6^ 293T-ACE2-TMPRSS2-mCherry cells were seeded in 25 cm^2^ flasks and infected the next day with 25 µL of frozen supernatant from the passaging. After 3–5 days, complete CPE was observed, and viruses were harvested by the collection of the supernatant and clarification by centrifugation. Aliquots were then made and stored at −80 °C until further usage. All viruses were titrated and sequence-confirmed prior to use, as discussed above for passaged viruses. Viruses retained analogous mutations as observed in the passaging, with the exception of P12-2G1, which had a low frequency of T304I during the passaging and this mutation was lost (the dominant S144A mutation was retained). Frequencies of mutations in each of these expanded passaged viruses are shown in Supplementary Data 6.

### Fitness assays

Growth of wild-type (with ORF3a and ORF7a deletions) and expanded passaged viruses was quantified over time to assay for fitness. One day prior to infection, A549-ACE2 cells were seeded in 12-well plates at a density of 2 × 10^5^ cells per well in 1 mL complete medium. The following day, cells were infected with 0.01 MOI of each virus. Two h post-infection, the supernatant was aspirated and cells were thoroughly washed with 1X PBS three times, and then 1 mL complete medium was added back to the wells. At 24, 48, and 72 h post-infection, 100 µL of the supernatant was harvested from each well and stored at −80 °C until further usage. To quantify the virus in the supernatant, 293T-ACE2-TMPRSS2-mCherry cells were seeded in 96-well plates at a density of 3 × 10^4^ cells per well in 100 µL complete medium and then were infected with 50 µL of the stored supernatant the next day. After 48 h, 50 µL of the supernatant was collected and viral RNA was extracted from all samples using the NucleoSpin 96 Virus kit (Macherey-Nagel) according to the manufacturer’s recommendations, and then quantified by qRT-PCR using TaqPath™ 1-Step RT-qPCR Master Mix, CG (Thermo Fisher Scientific) with SARS-CoV-2 RUO qPCR Primer & Probe Kit (IDT) on a 7500 Fast Dx Real-Time PCR Instrument (Applied Biosystems).

### Inhibition assays

Inhibition assays were conducted with the wild-type (without deletions), wild-type (with ORF3a and ORF7a deletions), or expanded passaged viruses to quantify drug inhibition. One day prior to infection, 293T-ACE2-TMPRSS2-mCherry cells were seeded in 96-well white-bottom plates at a density of 3 × 10^4^ cells per well in 100 µL complete medium. The following day, cells were infected with virus (normalized to amounts causing complete CPE, approximately 0.01 MOI) and treated with the appropriate inhibitor in a fivefold dilution series in 100 µL total. After an additional 48 h of incubation, CPE was quantified for all wells by CellTiter-Glo® 2.0 Cell Viability Assay (Promega) according to the manufacturer’s recommendations, using a CLARIOstar Plus (BMG Labtech) instrument with SMART Control software (BMG Labtech, version 6.10). Inhibition was calculated for each well relative to infected but untreated wells (0% inhibition) and uninfected wells (100% inhibition). IC_50_ values were derived by fitting a nonlinear regression curve to the data in GraphPad Prism version 10.6.0 (Dotmatics).

### Cell-based reporter assay for 3CL protease activity and drug inhibition

We adapted a previously reported cell-based reporter assay to evaluate the activity and inhibition of 3CL proteases. This assay utilizes FlipGFP^60^, a cleavage-activated GFP, which had been used by a number of groups to monitor SARS-CoV-2 3CL^pro^ activity^57–59^. While these prior studies have used microscopy as the readout, we sought to use flow cytometry to increase the throughput of our experiments.

We first constructed a set of plasmids necessary for our experimental design. All constructs in this study were generated by standard cloning approaches using Gateway cloning (Thermo Fisher Scientific), Gibson Assembly (NEBuilder HiFi DNA Assembly, New England Biolabs), and/or site-directed mutagenesis. Plasmids were amplified in NEB® 10-beta *E. coli* (for donor vectors; New England Biolabs), NEB Stable *E. coli* (for destination vectors; New England Biolabs), or ig® ccdB Resist™ *E. coli* (for constructs containing the *ccdB* gene; Intact Genomics). The plasmid backbones used were pDONR221 (Thermo Fisher Scientific), pLEX307-puro (Addgene, Catalog #41392, gift of D. Root), and pLEX307-miRFP680. The pDONR221 vector is a Gateway donor vector and the two pLEX307 vectors are Gateway destination vectors with constitutive expression of the gene of interest under an EF-1α promoter. pLEX307-puro additionally constitutively expresses puromycin N-acetyl-transferase for puromycin resistance, and pLEX307-miRFP680 additionally constitutively expresses miRFP680 as a fluorescent marker. pLEX307-miRFP680 was generated by replacing the puromycin N-acetyl-transferase gene in pLEX307-puro with miRFP680 from pmiRFP680-N1 (Addgene, Catalog #136557, gift of V. Verkhusha)^79^. miRFP680 was also cloned into pDONR221 then pLEX307-puro to generate pLEX307-puro-miRFP680 for strong constitutive expression of miRFP680 as a control plasmid. The FlipGFP sequence was synthesized as described^60^, with the cut site replaced with the SARS-CoV-2 3CL^pro^ cut site AVLQSGFR (Twist Biosciences). This insert was placed within both pLEX307-puro and pLEX307-miRFP680 to generate pLEX307-puro-FlipGFP-SARS-CoV-2-3CLcs and pLEX307-miRFP680-FlipGFP-SARS-CoV-2-3CLcs, respectively. The SARS-CoV-2 3CL^pro^ sequence was amplified from the CPER constructs described above, and cloned into pDONR221, then pLEX307-puro, generating pLEX307-puro-SARS-CoV-2-nsp5. This construct was then mutated to generate the following point mutants: C145A, T21I, L50F, S144A, E166A, E166V, and T304I.

Each of the 3CL proteases was then tested for their activity as measured by cleavage of FlipGFP. Lenti-X™ 293T cells were seeded one day prior to transfection in 96-well plates at a density of 3 × 10^4^ cells per well in 100 µL complete medium. The following day, cells were co-transfected with 20 ng of pLEX307-miRFP680-FlipGFP-SARS-CoV-2-3CLcs and 80 ng of the appropriate 3CL^pro^ plasmid per well using Lipofectamine™ 3000 (Thermo Fisher Scientific) according to the manufacturer’s recommendations. For compensation and gating, cells were transfected with pLEX307-puro-FlipGFP-SARS-CoV-2-3CLcs and wild-type 3CL^pro^ for FlipGFP signal only, and with pLEX307-puro-miRFP680 for miRFP680 signal only. One day post-transfection, cells were collected by trypsinization, washed once with complete medium, and then resuspended in complete medium and run on a LSR II (BD Biosciences) and analyzed on FlowJo (BD Biosciences, version 10). The gating strategy is shown in Supplementary Fig. 13. To calculate activity, each sample was first normalized to account for transfection variability by dividing the geometric mean fluorescent intensity (gMFI) of FlipGFP by the gMFI of miRFP680 within the miRFP680^+^ population. Activity was then calculated as a proportion of wild-type (100% activity) and C145A (0% activity), using the average values of wild-type and C145A.

Unexpectedly however, we observed that the relative activity of the mutant 3CL^pro^s was not in line with prior data (Supplementary Fig. 10a). Most notably, E166V had been previously shown by us and others to be significantly deficient in catalytic activity^61^, yet this mutant had robust activity exceeding wild-type in this assay format. We speculated that this may be in part due to the direct expression of the 3CL^pro^ not accounting for the requirement for it to be liberated from the polyprotein as in the viral life cycle, and we therefore sought to remake all of the 3CL^pro^ constructs in the form of nsp4-nsp5-nsp6 fusion proteins. For this purpose, we used a previously described design, in which the full-length nsp4 is fused to 3CL^pro^, then fused to the first six N-terminal residues of nsp6^80^. This nsp4-nsp5-nsp6 fusion protein was cloned into pDONR221, and then attempted to be cloned into pLEX307-puro. In our hands however, no successful clones could be recovered for any of the 3CL^pro^s in the original pLEX307-puro backbone despite repeated attempts. We finally isolated a successful construct with the nsp4-nsp5-nsp6 inserted into pLEX307-puro when a serendipitous *ori* mutation emerged, which significantly lowered the amount of plasmid that could be purified from a similar volume of *E. coli*. This suggested that this mutation lowered the copy number of the plasmid to a tolerable amount, and that there is potentially toxicity to *E. coli* associated with this fusion protein. Note that we have observed bacterial transposons emerging within the nsp4-nsp5-nsp6 genes during cloning, further supporting this possibility, and emphasizing the need to carefully confirm these plasmids prior to use. These constructs were then tested in an analogous manner as described above, and were found to reflect the expected relative activities associated with the mutations, affirming their usage in subsequent experiments (Fig. 4a). To confirm that the C145A mutant was catalytically-dead as we expected^81^, we co-transfected pLEX307-puro-NanoLuc, a plasmid which constitutively expresses NanoLuc, instead of a nsp4-nsp5-nsp6 plasmid with the FlipGFP reporter construct. C145A did not exhibit any activity when compared to NanoLuc being expressed instead (Supplementary Fig. 10b).

We then used these nsp4-nsp5-nsp6 fusion protein constructs for assessing the resistance profile of each of the mutant 3CL^pro^s against inhibitors. Lenti-X™ 293T cells were seeded and transfected with the FlipGFP and 3CL^pro^ constructs as described above. Concurrently with transfection, cells were also treated with the appropriate inhibitor in a fivefold dilution series in complete medium. One day post-transfection and drug treatment, cells were processed and analyzed by flow cytometry as described above. To calculate inhibition, each sample was first normalized for transfection by dividing the gMFI of FlipGFP by the gMFI of miRFP680 within the miRFP680^+^ population as above. Inhibition was then calculated for each sample relative to the respective wild-type or mutant 3CL^pro^ with no drug (0% inhibition) and 3CL^pro^ with C145A treated with an equivalent amount of drug (100% inhibition). IC_50_ values were derived by fitting a nonlinear regression curve to the data in GraphPad Prism version 10.6.0 (Dotmatics).

All of the plasmids generated for this assay have been deposited to Addgene (see Supplementary Data 8 for ID numbers and descriptions).

### Clinical mutation frequency analysis

The outbreak.info API^82^ (version 0.2.0) was used to retrieve the frequency of mutations from the Global Initiative on Sharing Avian Influenza Data (GISAID)^64^ database on Mar. 16, 2026 for each year between 2020 and 2024. Sequences were retrieved without specifying location (for global incidence) or by specifying the location to China, and mutations were searched with the getPrevalence() function.

### Materials availability

Plasmids generated and used for the cellular 3CL protease reporter assay have been deposited to Addgene under ID numbers 247684-247708 and 254471 and are described in Supplementary Data 8. Other materials used in this study will be made available under an appropriate Materials Transfer Agreement.

### Reporting summary

Further information on research design is available in the Nature Portfolio Reporting Summary linked to this article.

## Supplementary information

Supplementary Information

Description of Additional Supplementary Files

Supplementary Data 1

Supplementary Data 2

Supplementary Data 3

Supplementary Data 4

Supplementary Data 5

Supplementary Data 6

Supplementary Data 7

Supplementary Data 8

Reporting Summary

Transparent Peer Review file

## Source data

Source Data

## Data Availability

All experimental data are provided in the manuscript and Source Data are provided with this paper. Raw sequencing reads generated in this study have been deposited to NCBI SRA under BioProject accession number PRJNA1308603 and accession numbers for individual samples are listed in Supplementary Data 1. The 3CL^pro^ structures were downloaded from PDB under accession numbers 7JST^83^ (ligand-free), 8V4U^25^ (with ibuzatrelvir), 8IGX^22^ (with simnotrelvir), 8HBK^61^ (with ensitrelvir), and 7VH8^84^ (with nirmatrelvir). Source data are provided with this paper.

## Code availability

Sequencing data analyses were performed using the wf-artic pipeline (https://github.com/epi2me-labs/wf-artic) and LoFreq^76^. GISAID frequency analyses were performed using the outbreakinfo R package (https://outbreak-info.github.io/R-outbreak-info/)^82^. Some figures were generated using ggalluvial^78^ and/or ggplot2 (https://cran.r-project.org/web/packages/ggplot2/index.html) in R. Structures were visualized with UCSF ChimeraX^85^. All of the packages are publicly available.
