# Supplementary material for: Cross-resistance patterns in SARS-CoV-2 against 3CL protease inhibitors
Source: Nat Commun. 2026 May 19;17:6575. doi: 10.1038/s41467-026-73444-y (PMC13381909; doi:10.1038/s41467-026-73444-y)
Supplement: Supplementary file 1 — Supplementary Information [file 41467_2026_73444_MOESM1_ESM.pdf]

Name	Alternative names	Chemical structure
Nirmatrelvir	PF-07321332<br>Paxlovid (with ritonavir)	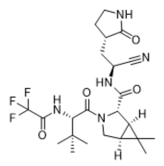
Ensitrelvir	S-217622<br>Xocova	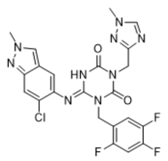
Atilotrelvir	GST-HG171	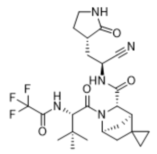
Ibuzatrelvir	PF-07817883	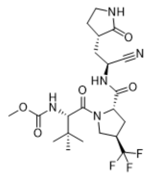
Leritrelvir	RAY1216	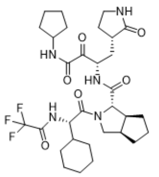
Simnotrelvir	SIM0417<br>Xiannuoxin (with ritonavir)	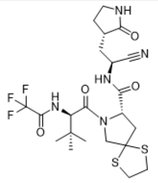

Supplementary Fig. 1. SARS-CoV-2 3CL^{pro} inhibitors used clinically or in development. Alternative names include their names during development and brand names.

a

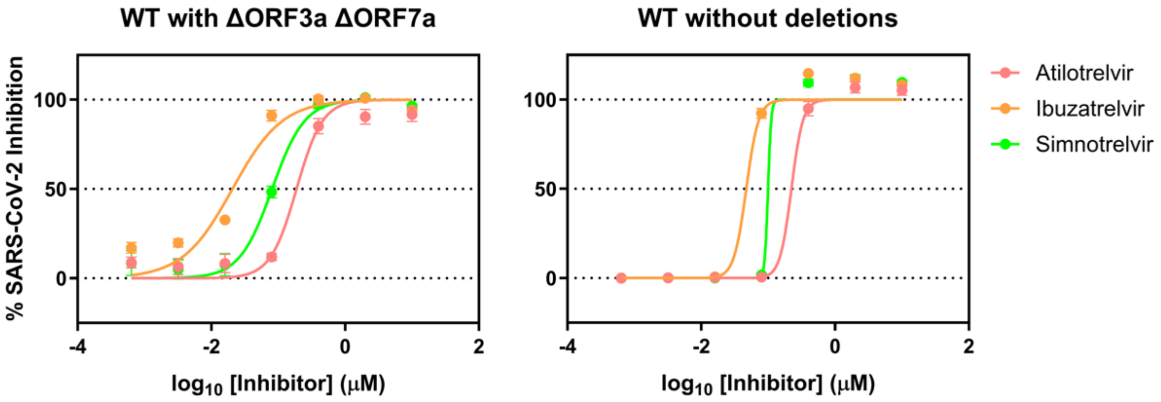

b

IC_{50} (μM) ± SD	Atilotrelvir	Ibuzatrelvir	Simnotrelvir
**WT with ΔORF3a ΔORF7a**	0.161 ± 0.039	0.026 ± 0.007	0.073 ± 0.011
**WT without deletions**	0.215 ± 0.011	0.035 ± 0.017	0.108 ± 0.012

c

	Atilotrelvir	Ibuzatrelvir	Simnotrelvir
**Fold change in IC_{50} (WT without deletions / WT with ΔORF3a ΔORF7a)**	1.34	1.36	1.48

Supplementary Fig. 2. Inhibition of wild-type SARS-CoV-2 and attenuated SARS-CoV-2. a, Inhibition curves of wild-type with ORF3a and ORF7a deletions (represented with Δ) or wild-type without deletions by atilotrelvir, ibuzatrelvir, and simnotrelvir in 293T-ACE2-TMPRSS2-mCherry cells. Representative curves from a single experiment from two biologically independent experiments are shown. Error bars denote mean ± SEM of three technical replicates. **b,** Raw IC_{50} values for inhibition of viruses. Values are shown as mean ± SD of two biologically independent experiments. **c,** Fold changes in mean IC_{50} values for inhibition of wild-type without deletions relative to wild-type with ORF3a and ORF7a deletions. Source data are provided as a Source Data file.

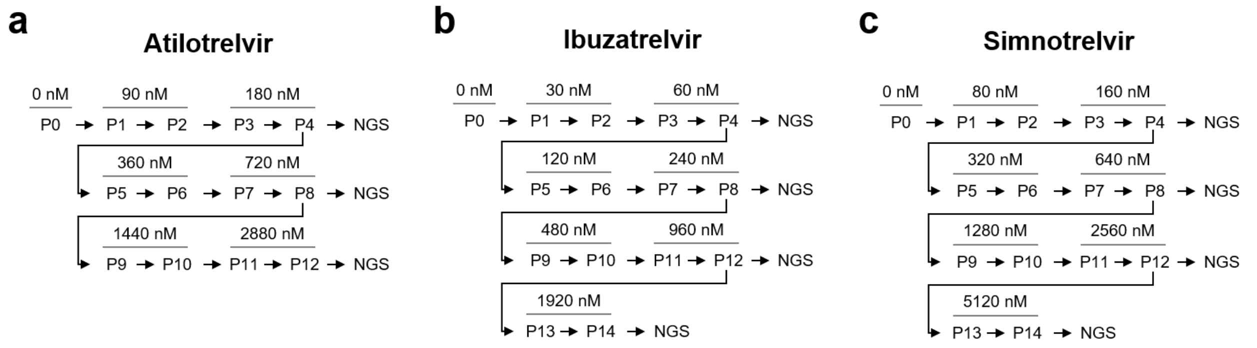

Supplementary Fig. 3. High-throughput viral passaging scheme to identify resistance-associated mutations. a-c, Passaging strategy for atilotrelvir (**a**), ibuzatrelvir (**b**), and simnotrelvir (**c**). Passaging for each $3CL^{pro}$ inhibitor was conducted with 180 independent replicates, with the dose doubled every other passage. Every fourth passage, as well as the terminal 14th passage for ibuzatrelvir and simnotrelvir, was sequenced to identify resistance-associated mutations. See Methods for further details.

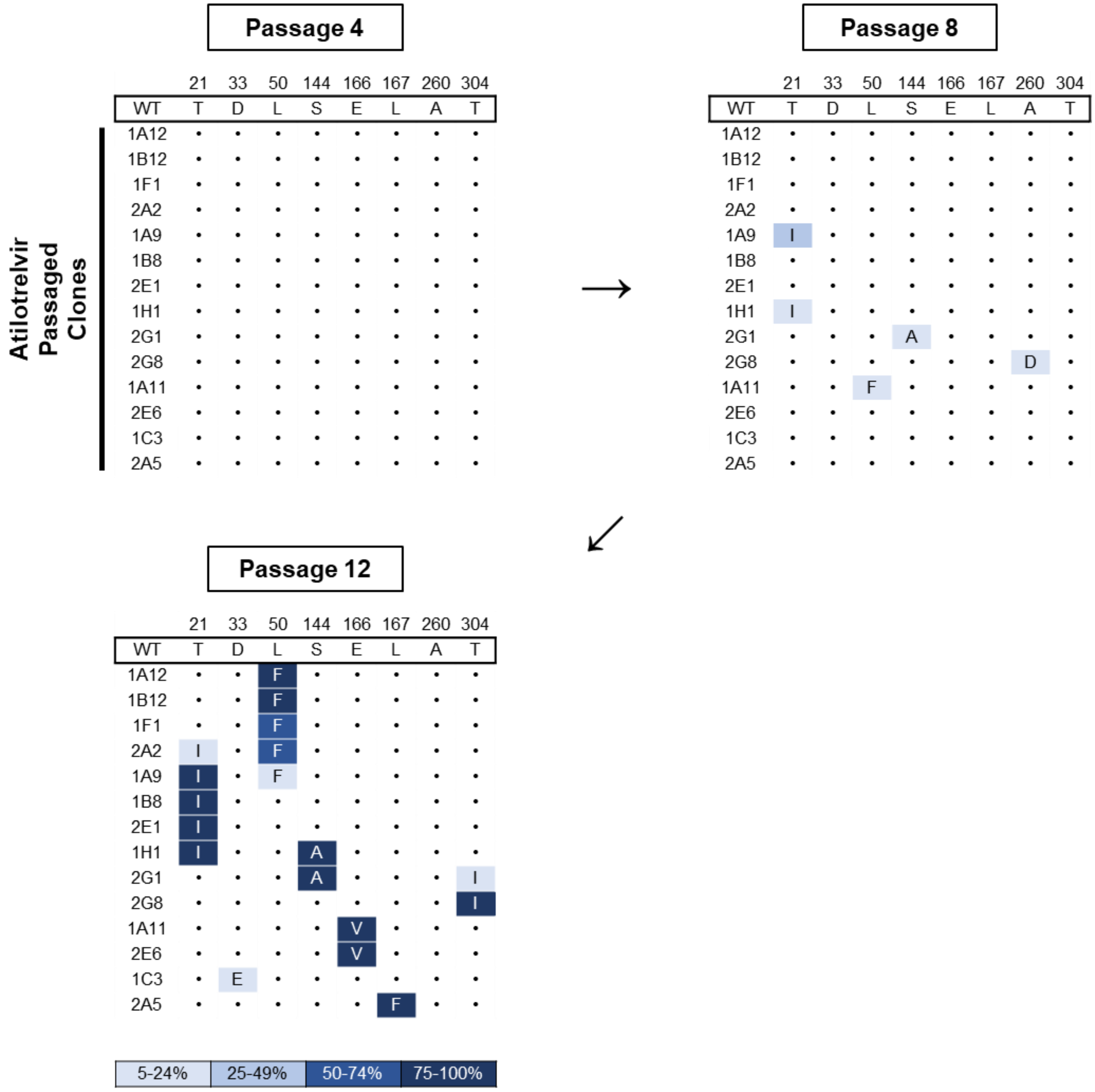

Supplementary Fig. 4. All 3CL^{pro} mutations which arose during passaging against atilotrelvir. Each well represents an independently passaged replicate. Dots indicate WT at that residue in that clone. Mutations are colored according to frequency (see Supplementary Table 2 for frequencies of each mutation in each clone). R222P is not shown as it was observed in untreated passaged viruses, suggesting it is a nonspecific cell-culture adaptation.

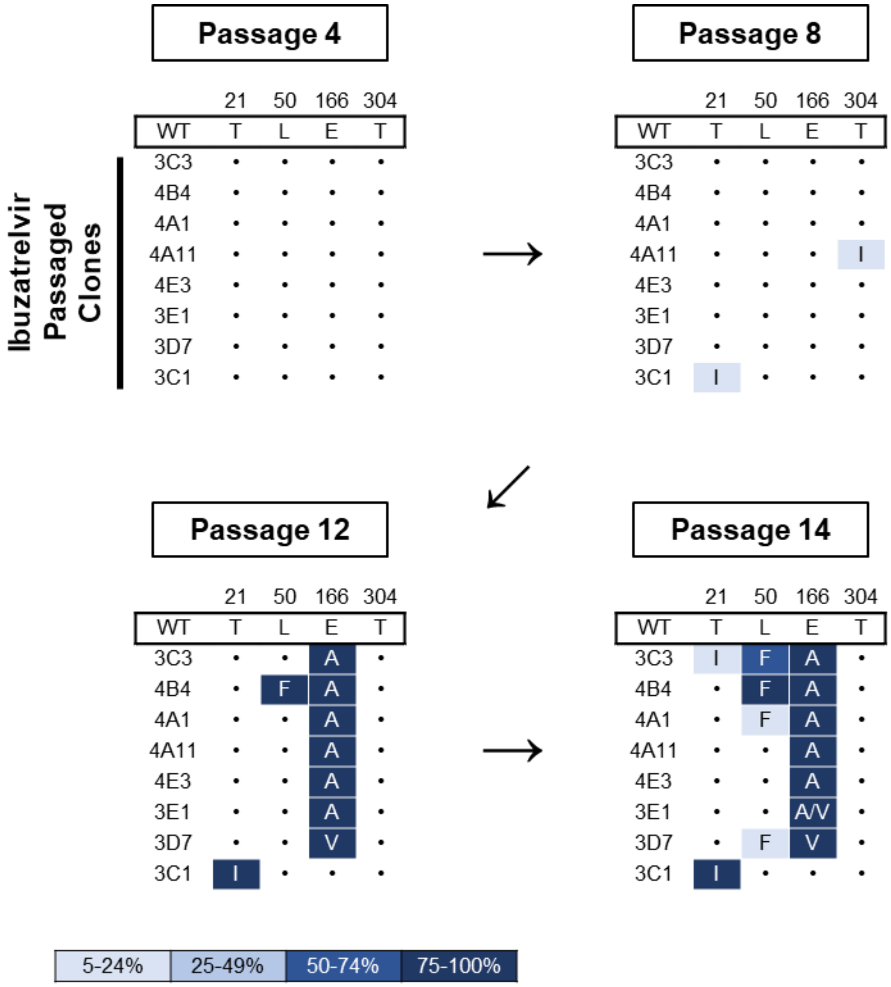

Supplementary Fig. 5. All 3CL^{pro} mutations which arose during passaging against ibuzatrelvir. Each well represents an independently passaged replicate. Dots indicate WT at that residue in that clone. Mutations are colored according to frequency (see Supplementary Table 2 for frequencies of each mutation in each clone). R222P is not shown as it was observed in untreated passaged viruses, suggesting it is a nonspecific cell-culture adaptation. A/V denotes a mixture of A and V.

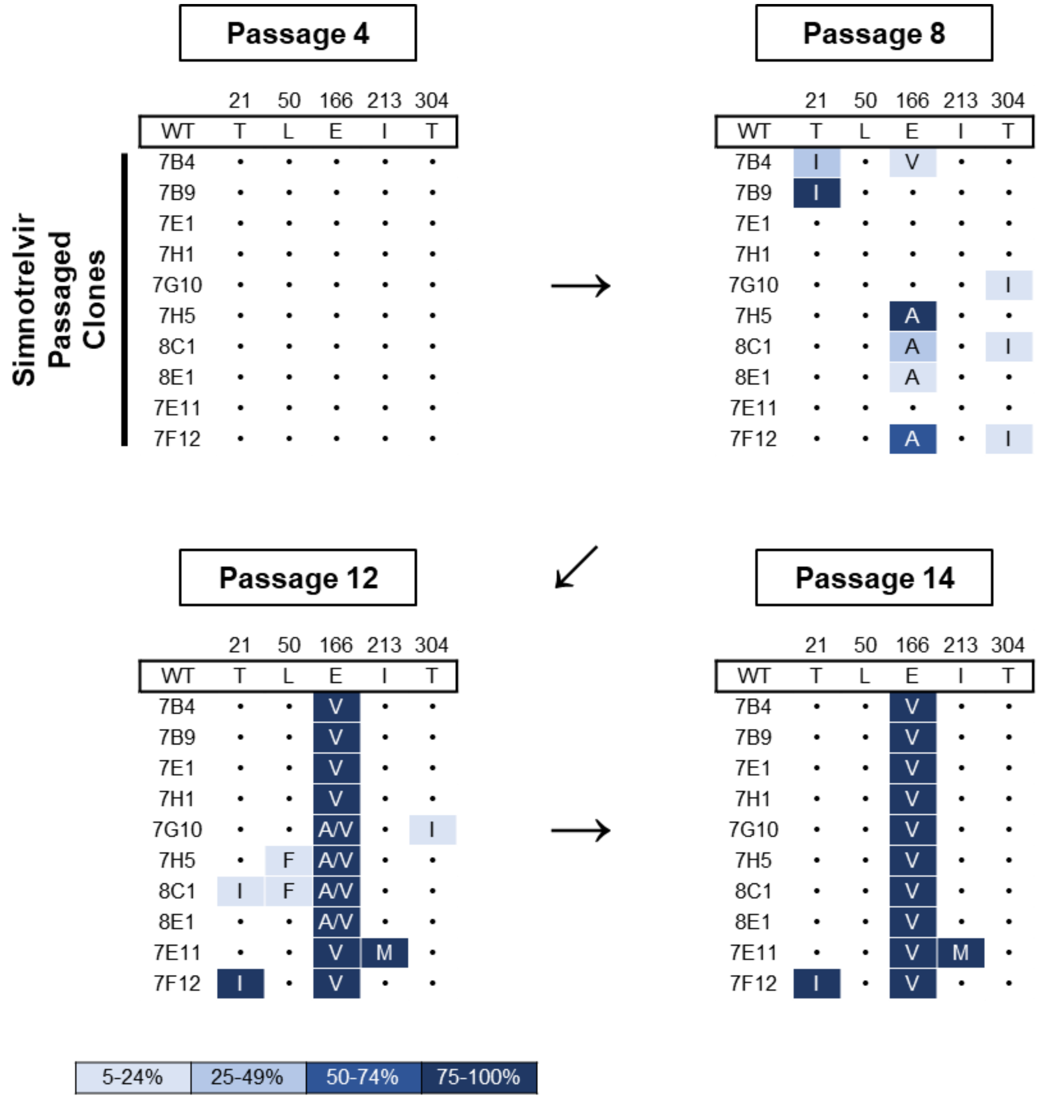

Supplementary Fig. 6. All 3CL^{pro} mutations which arose during passaging against simnotrelvir. Each well represents an independently passaged replicate. Dots indicate WT at that residue in that clone. Mutations are colored according to frequency (see Supplementary Table 2 for frequencies of each mutation in each clone). R222P is not shown as it was observed in untreated passaged viruses, suggesting it is a nonspecific cell-culture adaptation. A/V denotes a mixture of A and V.

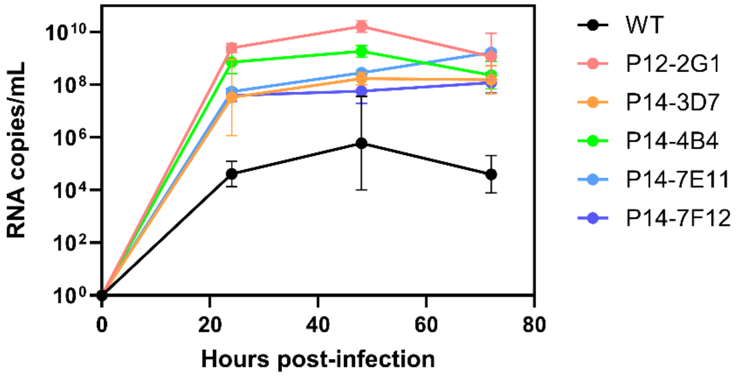

Supplementary Fig. 7. Fitness of passaged viruses. Growth assays were conducted for the passaged viruses in A549-ACE2 cells. The original unpassaged wild-type virus (with ORF3a and ORF7a deletions) was included as a reference. Values are shown as geometric mean ± geometric SD of three technical replicates. Source data are provided as a Source Data file.

Passaged against	nsp5 mutations	Clone	IC50 (μM) ± SD					
			Atilotrelvir	Ibuzatrelvir	Simnotrelvir	Ensitrelvir	Nirmatrelvir	Remdesivir
N/A	N/A	**WT**	0.159 ± 0.024	0.018 ± 0.004	0.103 ± 0.021	0.013 ± 0.001	0.016 ± 0.004	0.056 ± 0.008
Atilotrelvir	S144A	**P12-2G1**	0.917 ± 0.220	0.499 ± 0.265	1.29 ± 0.388	1.66 ± 0.408	0.227 ± 0.083	0.036 ± 0.006
Ibuzatrelvir	L50F + E166V	**P14-3D7**	>10.0 ± 0.000	>10.0 ± 0.000	>9.87 ± 0.135	0.631 ± 0.193	>10.0 ± 0.000	0.040 ± 0.020
	L50F + E166A	**P14-4B4**	3.61 ± 0.879	1.77 ± 0.104	3.53 ± 1.55	1.61 ± 0.085	1.1 ± 0.178	0.027 ± 0.003
Simnotrelvir	E166V + I213M	**P14-7E11**	>10.0 ± 0.000	3.78 ± 0.136	>10.0 ± 0.000	0.482 ± 0.015	>10.0 ± 0.000	0.018 ± 0.001
	T21I + E166V	**P14-7F12**	>10.0 ± 0.000	5.63 ± 2.14	>6.75 ± 3.25	0.318 ± 0.136	>10.0 ± 0.000	0.036 ± 0.016

Supplementary Fig. 8. Raw IC_{50} values for inhibition assays with passaged viruses. Raw IC_{50} values for inhibition of passaged viruses. Values are shown as mean ± SD of two biologically independent experiments. Passaged viruses were first sequenced to confirm their nsp5 mutations, which are listed in the second column (see Supplementary Table 6 for mutation frequencies). All clones are from the terminal passages against their respective compounds. Source data are provided as a Source Data file.

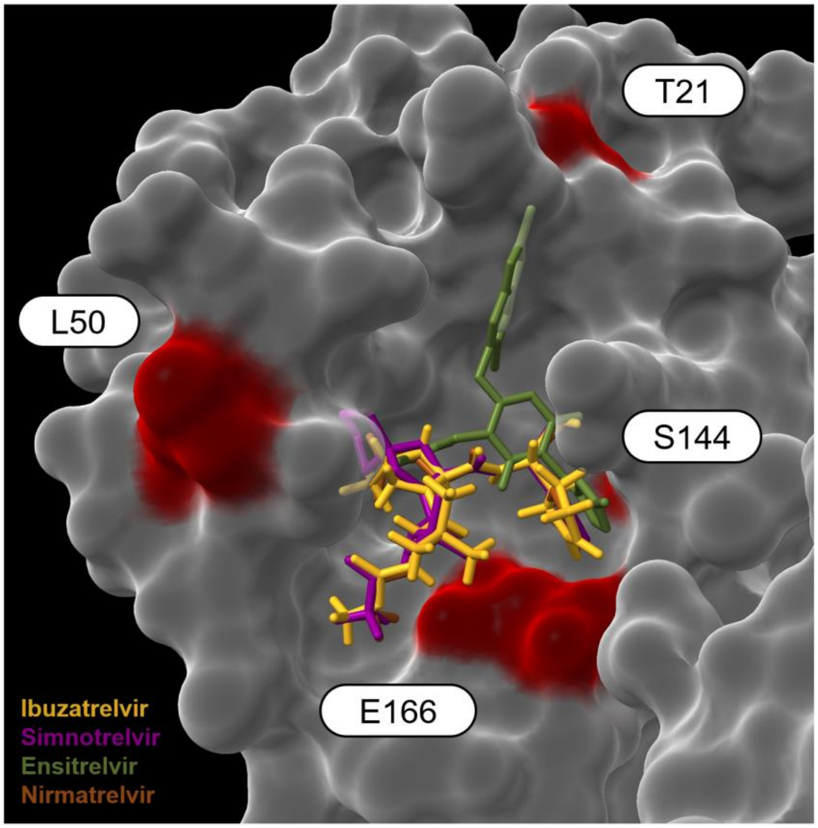

Supplementary Fig. 9. Structural overlay of 3CL^{pro} inhibitors. Ibuzatrelvir, simnotrelvir, ensitrelvir, and nirmatrelvir are shown within the 3CL^{pro} substrate binding site. Atilotrelvir is not shown as a structure was not available, but it has been previously modeled to likely bind in the same manner as nirmatrelvir[15]. Residues of interest are highlighted in red. Ibuzatrelvir is colored goldenrod, simnotrelvir is colored purple, ensitrelvir is colored dark olive green, and nirmatrelvir is colored saddle brown. 3CL^{pro} complexes were downloaded from PDB under accession codes 8V4U[25], 8IGX[22], 8HBK[61], and 7VH8[84], respectively.

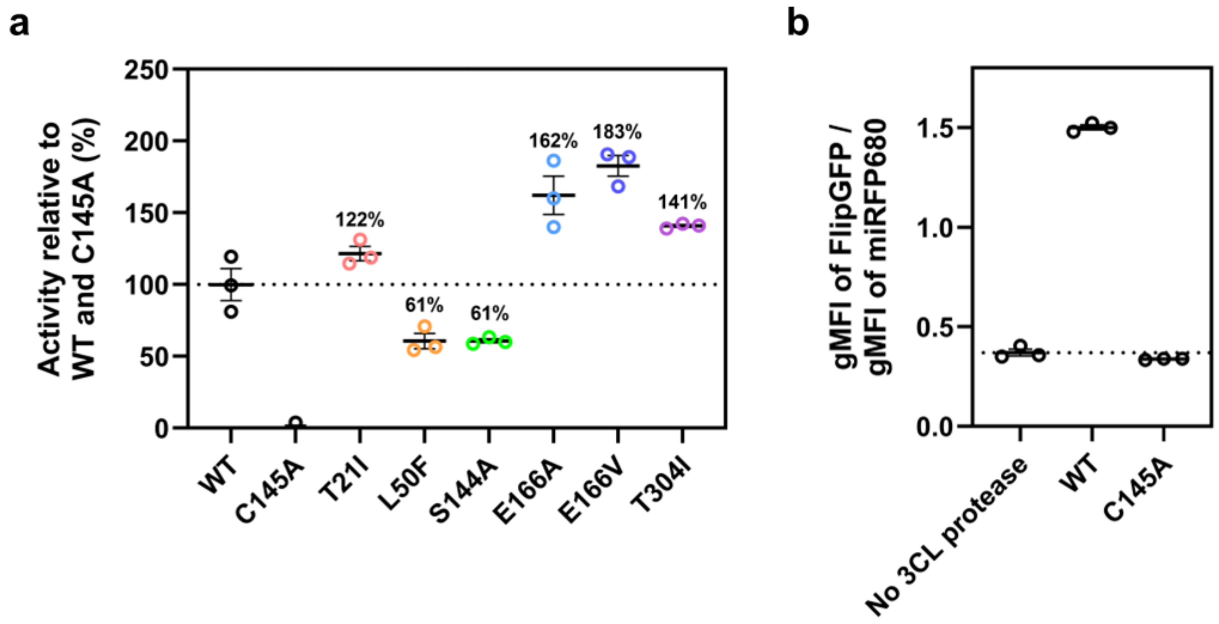

Supplementary Fig. 10. Activity of individual 3CL^{pro} mutants expressed as 3CL^{pro} alone and confirming C145A is inactive. a, Activity of wild-type or mutant 3CL proteases in the FlipGFP cellular reporter assay using 3CL^{pro}s expressed directly without fusion to nsp4 and nsp6 (see Methods for details). Representative results from a single experiment from two biologically independent experiments are shown. Data are shown as a scatter plot for three technical replicates, with error bars denoting mean ± SEM. Values above the points denote means. **b,** FlipGFP cellular reporter assay signal with wild-type or C145A mutant 3CL protease fused to nsp4 and nsp6, or without 3CL protease being expressed. Data are shown as a scatter plot for three technical replicates, with error bars denoting mean ± SEM. Source data are provided as a Source Data file.

3CL^pro	IC50 (μM) ± SD		
	**Atilotrelvir**	**Ibuzatrelvir**	**Simnotrelvir**
**WT**	0.377 ± 0.006	0.139 ± 0.065	0.470 ± 0.032
**T21I**	0.373 ± 0.033	0.294 ± 0.105	0.388 ± 0.277
**L50F**	5.76 ± 0.065	0.071 ± 0.024	0.666 ± 0.326
**S144A**	12.1 ± 8.30	2.32 ± 1.37	6.64 ± 1.34
**E166A**	>44.0 ± 8.54	>10.0 ± 0	>47.2 ± 3.94
**E166V**	>50.0 ± 0	>10.0 ± 0	>50.0 ± 0
**T304I**	0.446 ± 0.018	0.131 ± 0.013	0.317 ± 0.112

Supplementary Fig. 11. Raw IC_{50} values for inhibition assays in the FlipGFP cellular reporter assay. Raw IC_{50} values for inhibition of wild-type or mutant 3CL proteases. Values are shown as mean ± SD of two biologically independent experiments. Source data are provided as a Source Data file.

Mutation	Year	Number of sequences with mutation (Global)	Total sequences (Global)	Number of sequences with mutation (China)	Total sequences (China)
T21I	2020	283	581975	0	877
	2021	13183	6772276	5	1037
	2022	6246	6560157	7	8607
	2023	619	1215338	80	44290
	2024	41	368683	8	14325
L50F	2020	309	581975	1	877
	2021	3623	6772276	0	1037
	2022	1013	6560157	0	8607
	2023	122	1215338	4	44290
	2024	28	368683	5	14325
S144A	2020	0	581975	0	877
	2021	9	6772276	0	1037
	2022	6	6560157	0	8607
	2023	5	1215338	0	44290
	2024	0	368683	0	14325
E166A	2020	0	581975	0	877
	2021	3	6772276	0	1037
	2022	6	6560157	0	8607
	2023	1	1215338	0	44290
	2024	0	368683	0	14325
E166V	2020	2	581975	0	877
	2021	2	6772276	0	1037
	2022	15	6560157	0	8607
	2023	25	1215338	1	44290
	2024	2	368683	0	14325
T304I	2020	71	581975	0	877
	2021	649	6772276	0	1037
	2022	274	6560157	0	8607
	2023	29	1215338	0	44290
	2024	13	368683	0	14325

Supplementary Fig. 12. Frequencies of 3CL^{pro} mutants in clinical sequences. The number of sequences harboring the indicated mutations were searched for among all sequences or among sequences from China.

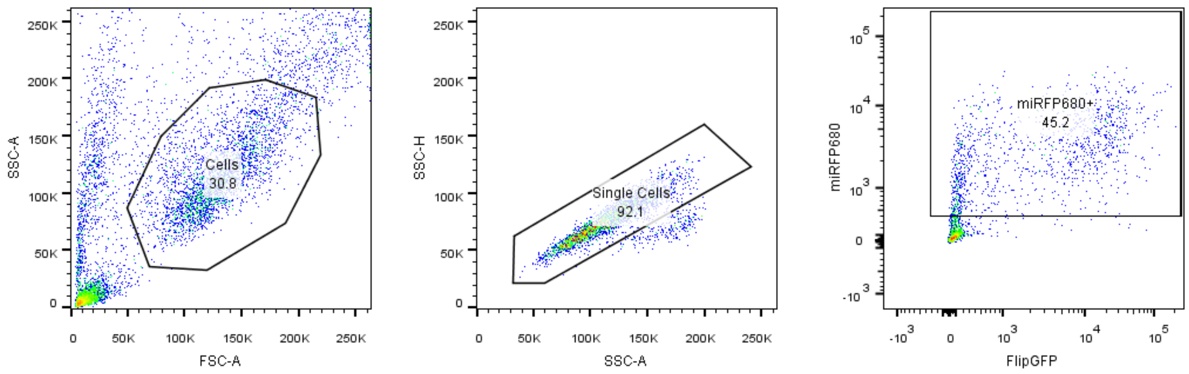

Supplementary Fig. 13. Gating strategy for cell-based 3CL^{pro} reporter assay by flow cytometry.
