# Supplementary material for: Cross-resistance patterns in SARS-CoV-2 against 3CL protease inhibitors
Source: Nat Commun. 2026 May 19;17:6575. doi: 10.1038/s41467-026-73444-y (PMC13381909; doi:10.1038/s41467-026-73444-y)
Supplement: Supplementary file 2 — Description of Additional Supplementary Files [file 41467_2026_73444_MOESM2_ESM.pdf]

Description of Additional Supplementary Files

Supplementary Data 1. Accession numbers for raw sequencing data. Data have been deposited to NCBI SRA under BioProject accession number PRJNA1308603.

Supplementary Data 2. Frequencies of nsp5 mutations across passages in viruses passaged against atilotrelvir, ibuzatrelvir, and simnotrelvir. All observed nsp5 mutations above the cutoff of 5% are shown.

Supplementary Data 3. Frequencies of nsp5 mutations in control wells. These clones were passaged for 14 passages without drug. All observed nsp5 mutations above the cutoff of 5% are shown.

Supplementary Data 4. Initial nsp5 mutations arising against 3CL^{pro} inhibitors. Only transitions resulting in T21I, L50F, S144A, E166A, E166V, or T304I were included, as these were the commonly observed mutations across compounds. See Methods for further details on this analysis. Data for nirmatrelvir is from our previous report[33].

Supplementary Data 5. Frequencies of nsp5 mutations at the conclusion of passaging. Only transitions resulting in T21I, L50F, S144A, E166A, E166V, or T304I were included, as these were the commonly observed mutations across compounds. Data for nirmatrelvir is from our previous report[33].

Supplementary Data 6. Frequencies of nsp5 mutations in expanded clones. These clones were expanded for use in further studies after the conclusion of passaging. All clones harbored analogous mutations as before expansion, with the exception of P12-2G1, which had a low frequency of T304I during the passaging and this mutation was lost (the dominant S144A mutation was retained). All observed nsp5 mutations above the cutoff of 5% are shown.

Supplementary Data 7. Fragment sequences and primers used for CPER. Fragments and primers used are analogous to those previously described[56], with modifications to fragment #9 and #10 to delete ORF3a and ORF7a.

Supplementary Data 8. Addgene ID numbers and descriptions for deposited plasmids.
