# Supplementary material for: Cross-resistance patterns in SARS-CoV-2 against 3CL protease inhibitors
Source: Nat Commun. 2026 May 19;17:6575. doi: 10.1038/s41467-026-73444-y (PMC13381909; doi:10.1038/s41467-026-73444-y)
Supplement: Supplementary file 11 — Reporting Summary [file 41467_2026_73444_MOESM11_ESM.pdf]

nature portfolio

Corresponding author(s): Sho Iketani

Last updated by author(s): Apr 14, 2026

Reporting Summary

Nature Portfolio wishes to improve the reproducibility of the work that we publish. This form provides structure for consistency and transparency in reporting. For further information on Nature Portfolio policies, see our Editorial Policies and the Editorial Policy Checklist.

Statistics

For all statistical analyses, confirm that the following items are present in the figure legend, table legend, main text, or Methods section.

n/a	Confirmed	
☐	☒	The exact sample size (*n*) for each experimental group/condition, given as a discrete number and unit of measurement
☐	☒	A statement on whether measurements were taken from distinct samples or whether the same sample was measured repeatedly
☒	☐	The statistical test(s) used AND whether they are one- or two-sided<br>*Only common tests should be described solely by name; describe more complex techniques in the Methods section.*
☒	☐	A description of all covariates tested
☒	☐	A description of any assumptions or corrections, such as tests of normality and adjustment for multiple comparisons
☐	☒	A full description of the statistical parameters including central tendency (e.g. means) or other basic estimates (e.g. regression coefficient) AND variation (e.g. standard deviation) or associated estimates of uncertainty (e.g. confidence intervals)
☒	☐	For null hypothesis testing, the test statistic (e.g. *F*, *t*, *r*) with confidence intervals, effect sizes, degrees of freedom and *P* value noted<br>*Give P values as exact values whenever suitable.*
☒	☐	For Bayesian analysis, information on the choice of priors and Markov chain Monte Carlo settings
☒	☐	For hierarchical and complex designs, identification of the appropriate level for tests and full reporting of outcomes
☒	☐	Estimates of effect sizes (e.g. Cohen's *d*, Pearson's *r*), indicating how they were calculated

Our web collection on statistics for biologists contains articles on many of the points above.

Software and code

Policy information about availability of computer code

Data collection	Luminescence was quantified on a CLARIOstar Plus (BMG Labtech) instrument with SMART Control software (BMG Labtech, version 6.10).
Data analysis	Sequencing data analyses were performed using the wf-artic pipeline (https://github.com/epi2me-labs/wf-artic; version 1.1.0) in EPI2ME (Oxford Nanopore Technologies, version 5.1.2) and LoFreq (version 2.1.5). GISAID frequency analyses were performed using the outbreakinfo R package (https://outbreak-info.github.io/R-outbreak-info/; version 0.2.0). Some figures were generated using ggalluvial (version 0.12.5) and/or ggplot2 (https://cran.r-project.org/web/packages/ggplot2/index.html; version 3.5.1) in R (version 4.4.1). Structures were visualized with UCSF ChimeraX (version 1.10.1). IC50 values were determined in GraphPad Prism (Dotmatics, version 10.6.0).

For manuscripts utilizing custom algorithms or software that are central to the research but not yet described in published literature, software must be made available to editors and reviewers. We strongly encourage code deposition in a community repository (e.g. GitHub). See the Nature Portfolio guidelines for submitting code & software for further information.

Data

Policy information about availability of data

All manuscripts must include a data availability statement. This statement should provide the following information, where applicable:

- Accession codes, unique identifiers, or web links for publicly available datasets
- A description of any restrictions on data availability
- For clinical datasets or third party data, please ensure that the statement adheres to our policy

All experimental data are provided in the manuscript and Source Data are provided with this paper. Raw sequencing reads generated in this study have been

deposited to NCBI SRA under BioProject accession number PRJNA1308603 and accession numbers for individual samples are listed in Supplementary Data 1. The 3CLpro structures were downloaded from PDB under accession numbers 7JST (ligand-free), 8V4U (with ibuzatrelvir), 8IGX (with simnotrelvir), 8HBK (with ensitrelvir), and 7VH8 (with nirmatrelvir).

Research involving human participants, their data, or biological material

Policy information about studies with human participants or human data. See also policy information about sex, gender (identity/presentation), and sexual orientation and race, ethnicity and racism.

Reporting on sex and gender	N/A; this study does not involve human participants, their data, or biological material
Reporting on race, ethnicity, or other socially relevant groupings	N/A; this study does not involve human participants, their data, or biological material
Population characteristics	N/A; this study does not involve human participants, their data, or biological material
Recruitment	N/A; this study does not involve human participants, their data, or biological material
Ethics oversight	N/A; this study does not involve human participants, their data, or biological material

Note that full information on the approval of the study protocol must also be provided in the manuscript.

Field-specific reporting

Please select the one below that is the best fit for your research. If you are not sure, read the appropriate sections before making your selection.

- [x] Life sciences
- [ ] Behavioural & social sciences
- [ ] Ecological, evolutionary & environmental sciences

For a reference copy of the document with all sections, see nature.com/documents/nr-reporting-summary-flat.pdf

Life sciences study design

All studies must disclose on these points even when the disclosure is negative.

Sample size	No sample size calculation was performed. We determined sample sizes from prior studies which allowed for replicable results (e.g., Iketani et al 2023 Nature).
Data exclusions	No data were excluded.
Replication	The passaging was conducted once. Viral inhibition assays and cellular reporter assays were repeated independently twice to verify the reproducibility of the findings. All replications produced consistent results.
Randomization	As this is an observational study, randomization is not relevant.
Blinding	As this is an observational study, investigators were not blinded.

Reporting for specific materials, systems and methods

We require information from authors about some types of materials, experimental systems and methods used in many studies. Here, indicate whether each material, system or method listed is relevant to your study. If you are not sure if a list item applies to your research, read the appropriate section before selecting a response.

Materials & experimental systems

n/a	Involved in the study	
x		Antibodies
	x	Eukaryotic cell lines
x		Palaeontology and archaeology
x		Animals and other organisms
x		Clinical data
	x	Dual use research of concern
x		Plants

Methods

n/a	Involved in the study	
x		ChIP-seq
	x	Flow cytometry
x		MRI-based neuroimaging

Eukaryotic cell lines

Policy information about cell lines and Sex and Gender in Research

Cell line source(s)	293T-ACE2-TMPRSS2-mCherry cells (HEK293T cells overexpressing human ACE2 and TMPRSS2) were obtained from BEI

	Resources (Catalog # NR-55293, gift of C. Weiss). Vero E6-TMPRSS2-T2A-ACE2 cells (Vero E6 cells overexpressing human TMPRSS2 and ACE2) were obtained from BEI Resources (Catalog #NR-54970, gift of B. Graham). Lenti-X™ 293T cells were purchased from Takara Bio (Catalog #632180). A549 cells were purchased from ATCC (Catalog #CCL-185). A549-ACE2 cells (A549 cells overexpressing human ACE2) were generated by transduction of A549 cells with lentivirus packaged using pLEX307-ACE2-blast (Addgene plasmid #158449), pMD2.G (Addgene plasmid #12259, gift of Didier Trono), and psPAX2 (Addgene plasmid #12260, gift of Didier Trono), followed by selection with 5 µg/mL blasticidin.
Authentication	Cell lines were purchased from authenticated vendors and confirmed by morphology.
Mycoplasma contamination	All cell lines tested mycoplasma negative.
Commonly misidentified lines (See ICLAC register)	No commonly misidentified lines were used in this study.

Dual use research of concern

Policy information about dual use research of concern

Hazards

Could the accidental, deliberate or reckless misuse of agents or technologies generated in the work, or the application of information presented in the manuscript, pose a threat to:

No	Yes	
☒	☐	Public health
☒	☐	National security
☒	☐	Crops and/or livestock
☒	☐	Ecosystems
☒	☐	Any other significant area

Experiments of concern

Does the work involve any of these experiments of concern:

No	Yes	
☒	☐	Demonstrate how to render a vaccine ineffective
☐	☒	Confer resistance to therapeutically useful antibiotics or antiviral agents
☒	☐	Enhance the virulence of a pathogen or render a nonpathogen virulent
☒	☐	Increase transmissibility of a pathogen
☒	☐	Alter the host range of a pathogen
☒	☐	Enable evasion of diagnostic/detection modalities
☒	☐	Enable the weaponization of a biological agent or toxin
☒	☐	Any other potentially harmful combination of experiments and agents

Precautions and benefits

Biosecurity precautions	All recombinant SARS-CoV-2 production, passaging, and infections were conducted by trained and vaccinated personnel in BSL-3 laboratories at Columbia University Irving Medical Center under procedures and guidelines approved by the Columbia University Institutional Biosafety Committee (IBC). Experiments involving recombinant viruses were only conducted utilizing an attenuated SARS-CoV-2 strain with double ORF3a and ORF7a deletions, as approved by NIAID prior to the initiation of the research.
Biosecurity oversight	Procedures were reviewed and approved by the Columbia University Institutional Biosafety Committee (IBC), and the use of attenuated SARS-CoV-2 for this study was approved by NIAID.
Benefits	Understanding the resistance profiles to these compounds informs their appropriate usage, such as in which patients these compounds should be used and how they could be used in combination and in salvage therapy. These data also help to provide guidance for future drug development.
Communication benefits	Communication of these results will allow for clinical surveillance and appropriate usage of the compounds, as well as provide insight into development of the next generation of protease inhibitors. As these described viruses remain susceptible to other therapeutic agents and arose naturally, we believe that communication of our data outweigh the risks.

Plants

Seed stocks	*Report on the source of all seed stocks or other plant material used. If applicable, state the seed stock centre and catalogue number. If plant specimens were collected from the field, describe the collection location, date and sampling procedures.*
Novel plant genotypes	*Describe the methods by which all novel plant genotypes were produced. This includes those generated by transgenic approaches, gene editing, chemical/radiation-based mutagenesis and hybridization. For transgenic lines, describe the transformation method, the number of independent lines analyzed and the generation upon which experiments were performed. For gene-edited lines, describe the editor used, the endogenous sequence targeted for editing, the targeting guide RNA sequence (if applicable) and how the editor was applied.*
Authentication	*Describe any authentication procedures for each seed stock used or novel genotype generated. Describe any experiments used to assess the effect of a mutation and, where applicable, how potential secondary effects (e.g. second site T-DNA insertions, mosiacism, off-target gene editing) were examined.*

Flow Cytometry

Plots

Confirm that:

☒ The axis labels state the marker and fluorochrome used (e.g. CD4-FITC).

☒ The axis scales are clearly visible. Include numbers along axes only for bottom left plot of group (a 'group' is an analysis of identical markers).

☒ All plots are contour plots with outliers or pseudocolor plots.

☒ A numerical value for number of cells or percentage (with statistics) is provided.

Methodology

Sample preparation	Lenti-X™ 293T cells were collected by trypsinization, washed once with complete medium, and then resuspended in complete medium and ran on a flow cytometer.
Instrument	LSR II (BD Biosciences)
Software	FlowJo (BD Biosciences, version 10)
Cell population abundance	Approximately 500 to 2,000 miRFP680+ cells were analyzed for each sample.
Gating strategy	Cells were gated by FSC/SSC, then singlets were gated by SSC-A/SSC-H. The gMFI of miRFP680 and FlipGFP were then calculated within the miRFP680+ population. Gates were determined by single color controls.

☒ Tick this box to confirm that a figure exemplifying the gating strategy is provided in the Supplementary Information.
