# Supplementary material for: Cross-resistance patterns in SARS-CoV-2 against 3CL protease inhibitors
Source: Nat Commun. 2026 May 19;17:6575. doi: 10.1038/s41467-026-73444-y (PMC13381909; doi:10.1038/s41467-026-73444-y)
Supplement: Supplementary file 12 — Transparent Peer Review file [file 41467_2026_73444_MOESM12_ESM.pdf]

nature portfolio

Peer Review File

Cross-resistance patterns in SARS-CoV-2 against 3CL protease inhibitors

Corresponding Author: Dr Sho Iketani

This file contains all reviewer reports in order by version, followed by all author rebuttals in order by version.

Version 0:

Reviewer comments:

Reviewer #1

(Remarks to the Author)
Here Chithelen and colleagues present a novel pipeline of assays to determine the emergence of protease resistance mutations in SARS CoV-2 3CLpro. Herein they use a novel high throughput pipeline and reverse genetics to determine the path to resistance of several protease inhibitors in use or in clinical trial. Overall, the study is well written and the assays reveal several convergent paths to protease resistance. In general terms it would be good to get a sense of what impact each resistance mutation would have if it were to appear in the community. For instance, would the mutations needed to become resistant sustain a significant drop in replicative fitness in an upper respiratory epithelial cell culture (ie. An in vitro surrogate for transmission). The approach looks very interesting and as such it would be good to have a simple schematic in the figures to map out the power of this vs older resistance based culture assays. Finally, do the authors have a sense on how fast the resistance would appear upon drug pressure and how this compares with other drugs where resistance appears rapidly versus slowly. The latter is often stop go criteria in Pharma drug development programs. A number of minor comments are as follows:

1-Line 39 State prevalence of severity here in the general vs immunocompromised populations.
2- Line 49 Maybe state “The effectiveness of those originally approved and tested in clinical trials has been compromised with the accumulation of specific Spike polymorphisms.”
3- Line 52. Several therapies remain in treatment or are in late clinical trials to determine efficacy that go beyond small molecule inhibitors. Best comment on small molecule inhibitors in this setting.
4- Line 208. Data in Extended figure 10 is hard to interpret for absolute numbers. Would it not be better to have the actual numbers in a table and then the total numbers submitted that month. Might also help to have a timeline of when the drugs were introduced into the clinic and importantly restrict analysis to the countries where they were available for use.
5- Line 2020: State this as Making the search for alternate therapies a priority with small molecule inhibitors part of this landscape of therapeutic options in the clinic.
6- Line 232: Would be good to have a schematic to show simply what this strategy is. Its sounds like a powerful means to look at resistance. It may also be good to understand why this approach is better than the standard approaches used in observing drug resistance. As someone outside of this field, it would be good to understand in simple terms the power of this approach over others.
7- Line 279: Outlining the challenges with the clinical used of certain protease inhibitors is important to state here. Paxlovid has a few interesting side effects and has DDIs with various heart medications (ie. Cannot be used in certain cases). So even continued refinement of drugs through drug discovery and combination use is of benefit to this field. In addition drug combination use has been shown to be very effective in many instances.

Reviewer #2

(Remarks to the Author)
This manuscript reports in vitro resistance pathways for three clinically relevant SARS-CoV-2 3CL protease inhibitors (atilotrelvir, ibuzatrelvir, and simnotrelvir) using large-scale viral passaging experiments. The authors demonstrate extensive cross-resistance among these and other 3CLpro inhibitors, including nirmatrelvir and ensitrelvir, with key mutations such as S144A, E166V, and E166A conferring broad resistance. Overall, this is a highly comprehensive and timely study that addresses an important and current issue; however, several points should be addressed.

The abstract contains references. As the abstract should be fully self-contained, all citations should be removed.

At line 128, the reference to “five compounds” seems inconsistent with the surrounding text and may be a typographical error (“four compounds”). Clarification would be helpful.

In Fig. 3b and Fig. 4c, the direction of the inequality symbols appears opposite to that described in the main text, which is confusing and should be clarified for consistency. In addition, the data could be presented more intuitively as “fold increase in IC50 relative to WT,” using only positive values. This would eliminate the need for the statement “Negative values denote resistance” and substantially improve clarity.

The catalytically-dead C145A mutant is mentioned only in the Methods as the 0% activity normalization control, but it is not clearly introduced in the Results section nor shown in Fig. 4a. The authors should explicitly mention this control in the Results and indicate it in the figure or legend.

While the experiments were performed using a USA-WA1/2020 backbone, a discussion of how these findings may translate to currently circulating variants would further strengthen the clinical relevance of the study.

Version 1:

Reviewer comments:

Reviewer #1

(Remarks to the Author)
The authors have addresses the comments I have made on the manuscript sufficiently. This work importantly addresses the resistance outcomes to two existing protease inhibitors and one in late clinical trials. The overlapping resistance profiles for the therapeutics studied herein are an important contribution to the clinical care of many & highlight the need for the development of future protease inhibitors with limited resistance overlap to those currently in use. It was not unlike that observed with Monoclonal antibodies, where one therapeutic antibody (Sotrovimab) was used far longer in the clinic, as the binding site did not overlap with the others in use. So in using their approach herein, future short listed drugs may be prioritised through their differing mode of action revealed in approaches like those listed herein.

Reviewer #2

(Remarks to the Author)
The authors have adequately addressed the reviewers' comments, improving the clarity and overall quality of the manuscript.

Open Access This Peer Review File is licensed under a Creative Commons Attribution 4.0 International License, which permits use, sharing, adaptation, distribution and reproduction in any medium or format, as long as you give appropriate

credit to the original author(s) and the source, provide a link to the Creative Commons license, and indicate if changes were made.
In cases where reviewers are anonymous, credit should be given to 'Anonymous Referee' and the source.
The images or other third party material in this Peer Review File are included in the article's Creative Commons license, unless indicated otherwise in a credit line to the material. If material is not included in the article's Creative Commons license and your intended use is not permitted by statutory regulation or exceeds the permitted use, you will need to obtain permission directly from the copyright holder.
To view a copy of this license, visit https://creativecommons.org/licenses/by/4.0/

We appreciate the positive feedback from the two reviewers and their suggestions which have strengthened our manuscript. Our responses to each of the comments are included below in blue. Changes within the manuscript file are highlighted in yellow.

Reviewer #1 (Remarks to the Author):

Here Chithelen and colleagues present a novel pipeline of assays to determine the emergence of protease resistance mutations in SARS CoV-2 3CLpro. Herein they use a novel high throughput pipeline and reverse genetics to determine the path to resistance of several protease inhibitors in use or in clinical trial. Overall, the study is well written and the assays reveal several convergent paths to protease resistance. In general terms it would be good to get a sense of what impact each resistance mutation would have if it were to appear in the community.

Thank you for all of your comments and feedback.

For instance, would the mutations needed to become resistant sustain a significant drop in replicative fitness in an upper respiratory epithelial cell culture (ie. An in vitro surrogate for transmission).

Thank you for this thoughtful suggestion. We set out to conduct growth assays with the passaged resistant viruses in a lung cell line as suggested. However, we do want to emphasize that for biosafety reasons, these viruses have purposely had two accessory proteins deleted (ORF3a and ORF7a), which are thought to have a variety of functions including downregulating the immune response. As a result, these viruses are quite attenuated and have difficulty growing in lung cell culture models which have strong immune responses. Nevertheless, after screening a number of cell lines and quantification approaches, we found that A549 cells (human lung adenocarcinoma) that we transduced to overexpress the SARS-CoV-2 receptor ACE2 could be used for growth assays with these viruses. These data are shown below:

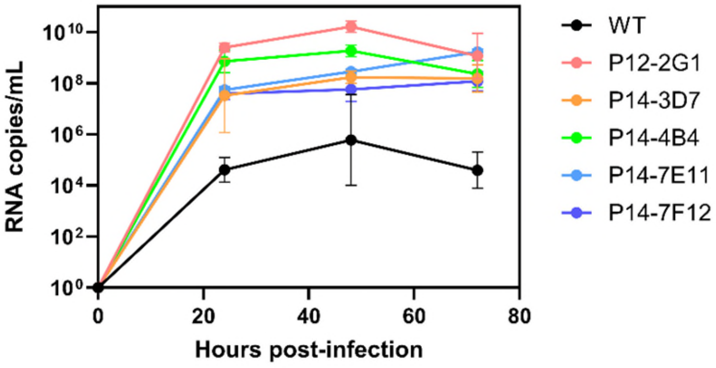

We observed that the five passaged viruses grew readily in these cells. As a reference we also included the original unpassaged wild-type virus with ORF3a and ORF7a deletions, which did not grow as well in this cell line. This likely reflects that the passaged viruses have adapted to in vitro cell culture over the course of passaging, which we also observed in our prior study (Iketani et al. 2023, Nature), in which we observed that an untreated yet equally passaged wild-type virus had improved growth kinetics over the original unpassaged wild-type virus.

We have incorporated the above data as **Extended Data Fig. 7** and have added the following to the text:

*"As these viruses had thus far been passaged only in 293T-ACE2-TMPRSS2-mCherry cells, we examined whether these viruses could grow in a human lung cell line. Growth assays in A549-ACE2 cells demonstrated that the passaged viruses could readily grow in this cell line as well, achieving greater titer than the original wild-type virus (with ORF3a and ORF7a double deletions) which had not been passaged (**Extended Data Fig. 7**). Such a difference may reflect adaptation to in vitro conditions, which we also observed in our prior study[33]."*

The approach looks very interesting and as such it would be good to have a simple schematic in the figures to map out the power of this vs older resistance based culture assays.

We greatly appreciate the interest in our high-throughput approach. We have added a simple schematic outlining the passaging strategy that was used for the three compounds as **Extended Data Fig. 3**:

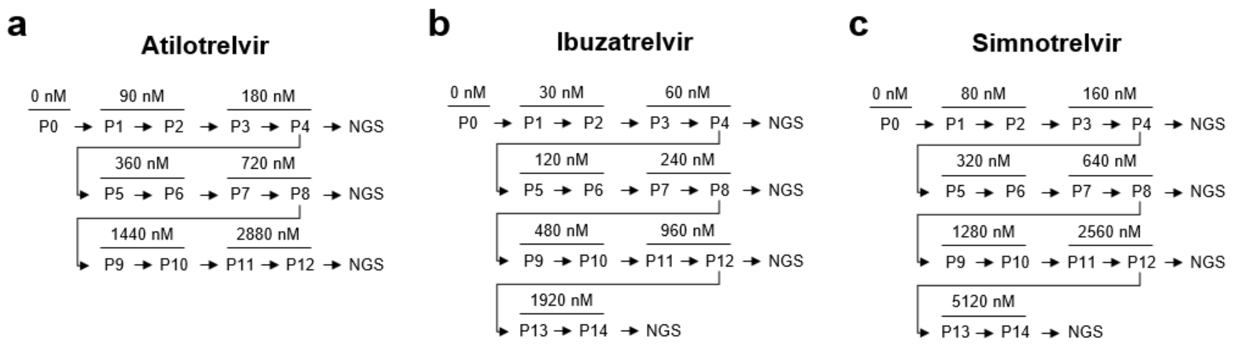

We have also added further discussion of the merits of the approach over more conventional approaches to the **Methods**, which now reads:

"*To identify resistance mechanisms, we adapted our previously reported strategy of high-throughput selection of SARS-CoV-2 resistance by blind volumetric passaging across a high number of replicates[33] (**Extended Data Fig. 3**). In this paradigm, we conduct infections in a 96 well-plate, with each well serving as an independent replicate. These replicates are treated with drug and passaged to new cells every three days, with drug doses doubled every other*

passage. As each well contains only a limited number of cells, there is an implicit bottleneck effect, which allows for both major and minor mutations to be observed, rather than dominant mutations overtaking the pool. In addition, the selection of resistance is expedited due to the use of a large number of replicates, which helps to curtail the effects of the stochasticity that can be present in infections. It is also appealing that this passaging approach does not involve the potentially laborious need for quantitation of virus in each replicate prior to passaging, as is typically done in standard passaging approaches to determine which replicates at which doses are moved forward. We previously observed that this high-throughput approach could select for resistant viral populations in nearly half of the number of passages as more conventional passaging strategies[33]."

In addition, we also added a sentence to the **main text** to note the benefits of this approach; we have left the bulk of the discussion of the benefits to the **Methods** given its length (the paragraph above). The text now reads:

"*We then used this virus to conduct high-throughput passaging against atilotrelvir, ibuzatrelvir, and simnotrelvir (**Fig. 1, Extended Data Fig. 3, 4, 5, and 6, and Supplementary Tables 1, 2, and 3**). Passaging was carried out with 180 replicates for each compound for 12 or 14 passages, employing an improved version of a scalable strategy that we had previously developed to identify nirmatrelvir resistance[33]. This usage of significantly more replicates than conventional passaging strategies can provide a more complete picture of the pathways to resistance as well as expedite the selection process (see **Methods** for details).*"

Finally, do the authors have a sense on how fast the resistance would appear upon drug pressure and how this compares with other drugs where resistance appears rapidly versus slowly. The latter is often stop go criteria in Pharma drug development programs.

This is a great point of consideration and we have been thinking about this question during our studies, given its importance for drug development as noted by the reviewer. It is a bit difficult to directly compare between the three compounds that we conducted passaging with here (or with nirmatrelvir in our prior study), as the doses that were used for passaging of each compound differ, and in addition, the clinically utilized dosage also differs between the compounds. Broadly in this current study we observed that the major resistance-associated mutations seemed to arise at a similar passage for the three compounds, but we do not think that this necessarily indicates whether resistance would arise quickly or not, and it is also important to note that in patients, we would not expect such escalating doses to be used, but rather a high dose to be immediately applied. An alternative approach that focuses specifically on this question, for example, by passaging at a fixed dose instead of escalating doses and seeing the rate of "breakthrough" events, would likely be needed to understand which compounds have higher or lower propensity for resistance. We are currently working on developing such approaches and hope to present them in a future publication.

A number of minor comments are as follows:

1-Line 39 State prevalence of severity here in the general vs immunocompromised populations.

Thank you for the suggestion. Unfortunately, it is difficult to ascertain the prevalence of severity in the general population now, as most mild cases are no longer reported or tracked. Cases are also increasingly not being reported or tracked even in severe cases and/or immunocompromised populations. It is known however, that rates of severity among immunocompromised populations is variable and dependent on the degree/type of immune suppression, underlying comorbidities, vaccine status, and/or treatment usage, among other factors. For example, a large retrospective cohort study in England found that the incidence of severe COVID-19 requiring hospitalization ranged from 0.1% to 13.1% of the overall subgroup (after grouping by differing immunocompromised status) (Evans et al. 2023, Lancet Reg Health Eur). Notably however, the authors noted that "all immunocompromised groups remained at increased risk of severe COVID-19 outcomes", regardless of the subgroup.

We have modified this sentence to note that prevalence of severity is dependent on a multitude of factors and added the above reference. We have avoided noting an exact prevalence given the wide range and the lack of recent testing/estimates of prevalence.

The text now reads: "In particular, the millions of individuals that do not have a robust response to vaccination or infection, such as the immunocompromised population, are at risk, and certain subgroups are particularly at high risk[2-7]."

2- Line 49 Maybe state "The effectiveness of those originally approved and tested in clinical trials has been compromised with the accumulation of specific Spike polymorphisms."

We have modified this sentence as suggested.

3- Line 52. Several therapies remain in treatment or are in late clinical trials to determine efficacy that go beyond small molecule inhibitors. Best comment on small molecule inhibitors in this setting.

We have modified this sentence to specify that we are referring only to direct-acting antiviral inhibitors and not other modalities for COVID-19 treatment.

4- Line 208. Data in Extended figure 10 is hard to interpret for absolute numbers. Would it not be better to have the actual numbers in a table and then the total numbers submitted that month. Might also help to have a timeline of when the drugs were introduced into the clinic and importantly restrict analysis to the countries where they were available for use.

Thank you for the suggestion. We have modified the data in this figure to a table with absolute numbers for each mutation and total sequences submitted in that year as suggested (now

Extended Data Fig. 12). As atilotrelvir and simnotrelvir are currently only authorized for usage in China, we have also filtered and added columns for sequences from China; however, we note that the number of sequences deposited to GISAID from China are low and it is therefore a bit difficult to understand trends related to the approvals of these compounds. Ibuzatrelvir is still in Phase 3 clinical trials and not yet available.

5- Line 2020: State this as Making the search for alternate therapies a priority with small molecule inhibitors part of this landscape of therapeutic options in the clinic.

We have incorporated this statement as suggested.

6- Line 232: Would be good to have a schematic to show simply what this strategy is. Its sounds like a powerful means to look at resistance. It may also be good to understand why this approach is better than the standard approaches used in observing drug resistance. As someone outside of this field, it would be good to understand in simple terms the power of this approach over others.

Thank you for this suggestion, as noted above, we have added a schematic and modified the main text as well as the Methods to further clarify the strengths of this approach.

7- Line 279: Outlining the challenges with the clinical used of certain protease inhibitors is important to state here. Paxlovid has a few interesting side effects and has DDIs with various heart medications (ie. Cannot be used in certain cases). So even continued refinement of drugs through drug discovery and combination use is of benefit to this field. In addition drug combination use has been shown to be very effective in many instances.

We very much agree that further inhibitors are undoubtedly needed. We have added a note for the need for the consideration of contraindications and side effects, which can limit the usage of current inhibitors, to the introduction, where it felt more appropriate.

The modified text now reads: "*These drugs have demonstrated clinical benefits and continue to be routinely employed as frontline therapies for COVID-19, although careful consideration of potential contraindications as well as the possibility of side effects are needed, which can limit their usage*[23,24]."

Reviewer #2 (Remarks to the Author):

This manuscript reports in vitro resistance pathways for three clinically relevant SARS-CoV-2 3CL protease inhibitors (atilotrelvir, ibuzatrelvir, and simnotrelvir) using large-scale viral

passaging experiments. The authors demonstrate extensive cross-resistance among these and other 3CLpro inhibitors, including nirmatrelvir and ensitrelvir, with key mutations such as S144A, E166V, and E166A conferring broad resistance. Overall, this is a highly comprehensive and timely study that addresses an important and current issue; however, several points should be addressed.

Thank you for the positive comments and careful suggestions.

The abstract contains references. As the abstract should be fully self-contained, all citations should be removed.

We have removed the references as suggested.

At line 128, the reference to “five compounds” seems inconsistent with the surrounding text and may be a typographical error (“four compounds”). Clarification would be helpful.

Thank you for catching this typo, we have corrected this.

In Fig. 3b and Fig. 4c, the direction of the inequality symbols appears opposite to that described in the main text, which is confusing and should be clarified for consistency. In addition, the data could be presented more intuitively as “fold increase in IC50 relative to WT,” using only positive values. This would eliminate the need for the statement “Negative values denote resistance” and substantially improve clarity.

Thank you for the suggestion, we have amended both figures as suggested.

The catalytically-dead C145A mutant is mentioned only in the Methods as the 0% activity normalization control, but it is not clearly introduced in the Results section nor shown in Fig. 4a. The authors should explicitly mention this control in the Results and indicate it in the figure or legend.

Thank you for noticing this issue. The C145A mutant is shown in Fig. 4a, but the bar and error bar were too small to see in this panel (as it is normalized to zero). To remedy this issue, we have added data for an experiment directly comparing C145A versus not having any 3CL protease expressed (NanoLuc was expressed instead, to equilibrate plasmid DNA input). The wild-type 3CL protease was included as a positive control. As shown below, the C145A mutant has no additional signal compared to having no 3CL protease expressed, in stark contrast to the wild-type 3CL protease. These data have been added to the manuscript as **Extended Data Fig. 10a** and are discussed in the **Methods**. The NanoLuc plasmid that was used for this experiment has been deposited to Addgene.

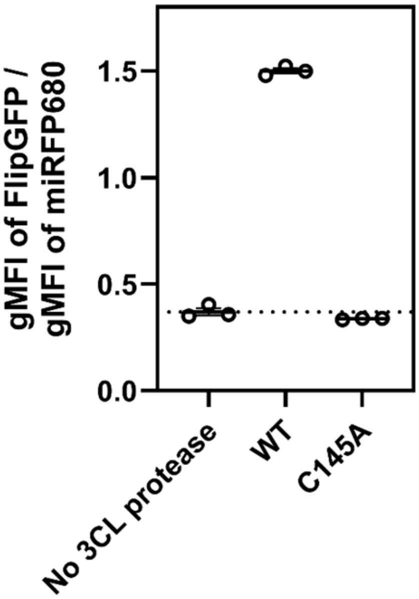

While the experiments were performed using a USA-WA1/2020 backbone, a discussion of how these findings may translate to currently circulating variants would further strengthen the clinical relevance of the study.

We agree that different variants may have alternative evolutionary routes. We have edited the Discussion on this point to further emphasize that current circulating variants have many mutations in their genome relative to the USA-WA1/2020 backbone used, and that other studies have found that this may impact the mutations observed.

The text now reads: "*It should also be pointed out that we have only conducted the passaging in one cell line, and that we have used the original USA-WA1/2020 strain as the base of our experiments. It is possible that some of the results may differ in an alternative cell type, or with the current variants, which harbor P132H in the 3CL^{pro} gene as well as numerous additional mutations in other genes throughout their genomes. Other studies have noted that the fitness impact and emergence of 3CLpro mutations can be impacted by the SARS-CoV-2 backbone*[34,69]*. Additional studies will be needed to investigate these prospects*."
